# Perfectionism increases among Chinese college students: a cross-temporal meta-analysis

**DOI:** 10.1186/s40359-026-05169-y

**Published:** 2026-07-13

**Authors:** Ruimeng Wang, Yuxiang Luan

**Affiliations:** 1 School of Human Resources, Guangdong University of Finance & Economics, Guangzhou, China; 2 Business School, Sichuan University, Chengdu, China

**Keywords:** Perfectionism, Cross-temporal meta-analysis, College students, Personality trait, Modernization

## Abstract

Since China’s accession to the WTO, the accelerated pace of modernization, which was characterized by rapid economic growth and profound societal transformations, has significantly intensified interpersonal competition. However, it remains unclear whether this swift development contributes to an increase in perfectionism, a personality trait closely linked to competition. Using studies identified through searches of CNKI, Wanfang, Web of Science, and Scopus, this study employs a cross-temporal meta-analytic approach, synthesizing 355 effect sizes from 74 studies (*N* = 37,279), to systematically investigate trends in perfectionism and its core dimensions among mainland Chinese college students from 2002 to 2022. Key findings reveal a statistically significant upward trajectory in FMPS-assessed overall perfectionism levels and its two critical dimensions, namely, concern over mistakes and doubts about actions. Three key modernization indicators (divorce rate, per capita GDP, and urbanization rate) demonstrate significant positive correlations with perfectionism, while family size shows a negative correlation. These findings provide evidence elucidating the association between China’s rapid modernization and college students’ FMPS-assessed perfectionism and offer evidence-based references for policy formulation and psychological intervention practices.

**Supplementary Information:**

The online version contains supplementary material available at https://doi.org/10.1186/s40359-026-05169-y.

## Introduction

The traditional Chinese saying, "Gold can't be pure, and people can't be perfect", suggests that individuals should not be expected to be flawless. As a multidimensional personality trait, perfectionism is defined as the combination of setting excessively high personal standards and harsh self-evaluation [12], and has garnered increasing academic attention in recent years [37]. Empirical evidence indicates that perfectionism is linked to a range of negative mental health outcomes. For instance, a longitudinal meta-analysis found a positive association between perfectionism and anxiety [38], while cross-sectional meta-analyses have linked it to poorer sleep quality [40], heightened burnout [18], and lower academic performance [28].

Over the past four decades, China's modernization has reshaped its social structure, driving notable shifts in individual personality traits [3]. Evidence shows increases in employees’ core self-evaluations [26], changes in Big Five traits among college students [30], and rising levels of loneliness [47]. Given that personality is strongly shaped by social context [33], and perfectionism is linked to competitive pressures [9], it is likely sensitive to such macro-level transformations. The spread of competitive culture in China [32], combined with college students’ high sensitivity to social change [48], may contribute to generational shifts in perfectionism. While prior research has documented rising perfectionism among American, Canadian, and British college students in the context of neoliberal competition [10], longitudinal evidence from China remains limited.

This study employs a cross-temporal meta-analysis to systematically investigate the trajectory of perfectionism among Chinese college students (2002–2022). This cohort was selected for three key reasons. The first one is to reveal the psychological imprints of modern competition, where post-WTO accession economic surges have intensified societal competition, making perfectionism a salient psychological tendency among successive cohorts of college students. The second is to target a persistent influence source, as college students' perfectionism exhibits relative stability, whose cumulative effects will shape mate selection standards and fertility decision-making. The third is to reduce age heterogeneity across samples. Because the included participants were all college students, score differences across years are more likely to reflect historical differences across successive student cohorts rather than large age-related differences [9].

Research on psychological change should address two core questions: the trend (how) and the mechanism (why) of change [4]. Building on this framework, the present study not only maps the developmental trajectory of perfectionism among Chinese college students but also investigates its association with modernization-related indicators, offering empirical insight into the drivers of change. As the first diachronic analysis of perfectionism in mainland Chinese college students, this study responds to researchers’ call for research on psychological shifts in China [3].

The contribution of this work lies in three key areas. First, it bridges macro-level socioeconomic indicators with micro-level psychological traits, enriching our understanding of perfectionism’s underlying causes. Second, it extends the psychological map of modernization by elucidating how macro social processes shape individual traits. Third, this study offers a practical foundation for college mental health services by highlighting rising trends in perfectionism and their potential risks. Because perfectionism, particularly its maladaptive dimensions, is associated with anxiety, depression, and other psychological difficulties [37, 38], identifying temporal increases in perfectionism and its risk-related dimensions among college students may provide a reference value for screening, early identification, and targeted prevention in university mental health services. As Smith et al. [37] note, understanding perfectionists’ adaptive mechanisms is critical for improving social support systems and fostering better integration.

### Influencing factors of perfectionism

Early research conceptualized perfectionism as an excessive pursuit of high standards [19]. Since the 1990 s, it has been widely recognized as a multidimensional personality trait [37]. For example, perfectionism is conceptualized as a self-oriented multidimensional construct comprising concern over mistakes, high personal standards, doubts about actions, perceived parental criticism and expectations, and organization [12]. Concern over mistakes reflects a dichotomous evaluation of one’s performance, viewing anything less than perfection as failure. Personal standards capture the tendency to set unrealistically high goals. Doubts about actions indicate uncertainty regarding the quality of one's behavior. Perceived parental criticism and expectations represent individuals’ internalized perceptions of harsh judgment and unrealistic demands from parents. The dimension of organization is considered relatively adaptive, reflecting an emphasis on precision and orderliness. Despite variations in dimensional frameworks, these perspectives converge on the view that the essence of perfectionism lies in an overcommitment to flawlessness and heightened sensitivity to failure, whether driven by internal motivation, interpersonal expectations, or social norms [12, 17].

While research on perfectionism has primarily examined its outcomes, less is known about its antecedents [9]. Evidence suggests that perfectionism is shaped by both genetic and environmental factors. Twin studies show moderate heritability, with genetic influences accounting for 23–30% of variance in self-oriented perfectionism and 39–42% in socially prescribed perfectionism [23]. Environmental contributors include psychologically controlling parenting, especially those emphasizing dependency or achievement, and parental achievement goals, all of which have been linked to the development of perfectionistic tendencies in children [1, 39].

Culturally, Curran et al. [10] found that perfectionism continued to rise among college students in the United States, Canada, and the United Kingdom over the past 35 years, with particularly marked increases since the early 2000 s in socially prescribed perfectionism, which reflects perceived external demands for perfection and perfectionistic concerns. They further linked these temporal changes to macroeconomic conditions, showing associations with GDP per capita and income inequality. However, these findings are based on Western samples, and the proposed macro-level explanations may not readily apply to China’s perfectionism trends.

### The modernization development and shifts in perfectionism among Chinese college students

Since China's reform and opening-up, particularly its 2001 WTO accession as a pivotal milestone, transformative socioeconomic changes have occurred. By 2024, China maintained its position as the world's second-largest economy (GDP: 134.9 trillion yuan), with per capita disposable income reaching 41,314 RMB and urbanization exceeding 67%. This rapidly evolving context may significantly impact individuals’ psychological traits.

Greenfield's [13] theoretical framework elucidates modernization's influence on perfectionism through a four-tiered pathway: 1) shifting sociodemographic characteristics reshape cultural values,2) new values alter learning environments (e.g., parenting, education); 3) modified environments affect cognitive/affective development, ultimately shaping personality traits like perfectionism.

Accordingly, we select urbanization, GDP per capita, household size, and divorce rates as indicators of China's modernization—variables whose changes may be associated with cultural shifts and proximal learning environments, thereby providing a broader context for understanding changes in perfectionism.

China’s rapid modernization, which is characterized by urbanization, economic growth, and shifting social dynamics, has been accompanied by increasing competition and a faster pace of life. In this context, cultural values have shifted toward individualism and achievement, while collectivist ideals have weakened [16]. Changes in educational environments further reflect this shift, with parents and schools placing greater emphasis on academic and career success [13]. Over time, these external demands may provide a possible context in which perfectionistic tendencies develop.

Moreover, weakened social bonds may heighten perfectionism [13]. While social support buffers anxiety amid competitive pressures [34], China's social transformations—manifested in shrinking households and diminished familial support [46]—have eroded such connections. Insufficient support may be related to stronger needs for social validation and heightened fear of failure, thereby providing a possible context for higher levels of perfectionism.

### The present study

This study has two objectives: first, to delineate the trajectory of perfectionism among Chinese mainland college students during the 2002–2022 period through cross-temporal meta-analysis; second, to examine the predictive effects of four modernization indicators—urbanization rate, GDP per capita, household size, and divorce rate—on perfectionism and its dimensions. By focusing on these indicators, the present study provides evidence on the distal societal correlates of perfectionism change. These indicators were selected based on Greenfield's [13] model and empirical precedents. Urbanization was operationalized using urbanization rate [31], economic development by GDP per capita [26], and social relationships—particularly within the family, which play a key role in early adulthood—were captured through household size and divorce rate [31]. Prior research has linked long-term changes in divorce rate to socioeconomic development and broader modernization processes, and has treated divorce rate as a key indicator of marital and family change in China [29], [51]. In this sense, divorce rate represents a societal-level marker of modernization-related changes in marital stability and family relations. This indicator is theoretically relevant to perfectionism because it may reflect a broader weakening of family-based support and social bonds, which may increase individuals’ needs for social validation and fear of failure, thereby providing a possible context for higher levels of perfectionism.

Cross-temporal meta-analysis offers a methodological approach to address both research objectives. Unlike traditional meta-analyses, which typically synthesize effect sizes such as correlation coefficients (*r*) to estimate population parameters (*ρ*) while reducing sampling and measurement error [35], cross-temporal meta-analysis uses sample means (*M*) as outcome variables to examine changes in psychological constructs over time [43]. Each study included in the analysis represents a sample from a specific historical period, allowing researchers to trace temporal trends in psychological traits [21].

To address the first objective, we collected mean scores of perfectionism and its dimensions among mainland Chinese college students across different years and conducted a cross-temporal meta-analysis to assess longitudinal trends. To address the second, we employed weighted regression analyses to examine the predictive effects of four modernization-related indicators on perfectionism scores using publicly available national data.

## Methods

### Measurement

To analyze longitudinal trends in perfectionism among mainland Chinese college students, this study employed the Frost Multidimensional Perfectionism Scale (FMPS; [12] and its Chinese version [52]. As a widely recognized instrument for assessing perfectionism, the Chinese adaptation has undergone rigorous translation and cultural adjustment to ensure validity and applicability in the Chinese sociocultural context, allowing for reliable assessment of both overall perfectionism and its specific dimensions.

### Literature search

To comprehensively collect research literature on perfectionism in the Chinese context, this study conducted literature searches from multiple data sources. Chinese literature used the China National Knowledge Infrastructure and Wanfang databases, while English literature used the Web of Science and SCOPUS databases. The search included both journal articles and graduate theses/dissertations, including master’s and doctoral dissertations indexed in CNKI. The search keywords used in this study were "perfection" and "perfectionism". The search time ended in February 2024. In order to more accurately locate studies containing Chinese samples or backgrounds, "Chinese" or "China" was added as additional keywords when searching English literature.

### Inclusion and exclusion criteria

Studies were included in the meta-analysis if they met the following criteria: (a) The measure of perfectionism was the FMPS [12] or its Chinese version [52], (b) The study reported either total or subscale scores of perfectionism, expressed as raw totals or mean item scores (converted where necessary),(c) The sample consisted exclusively of college students from mainland China; studies involving non-college samples (e.g., adolescents, working adults) or overseas Chinese populations were excluded; (d) Sample size (*N*) was reported to allow for effect size weighting; (e) Only studies using the original 5-point Likert scale were included. A few studies (*k* = 2) using a 7-point scale were excluded to ensure measurement consistency, as response format may affect response style [50]. This follows the original FMPS design [12] and prior meta-analytic practice [45].

**Fig. 1:**
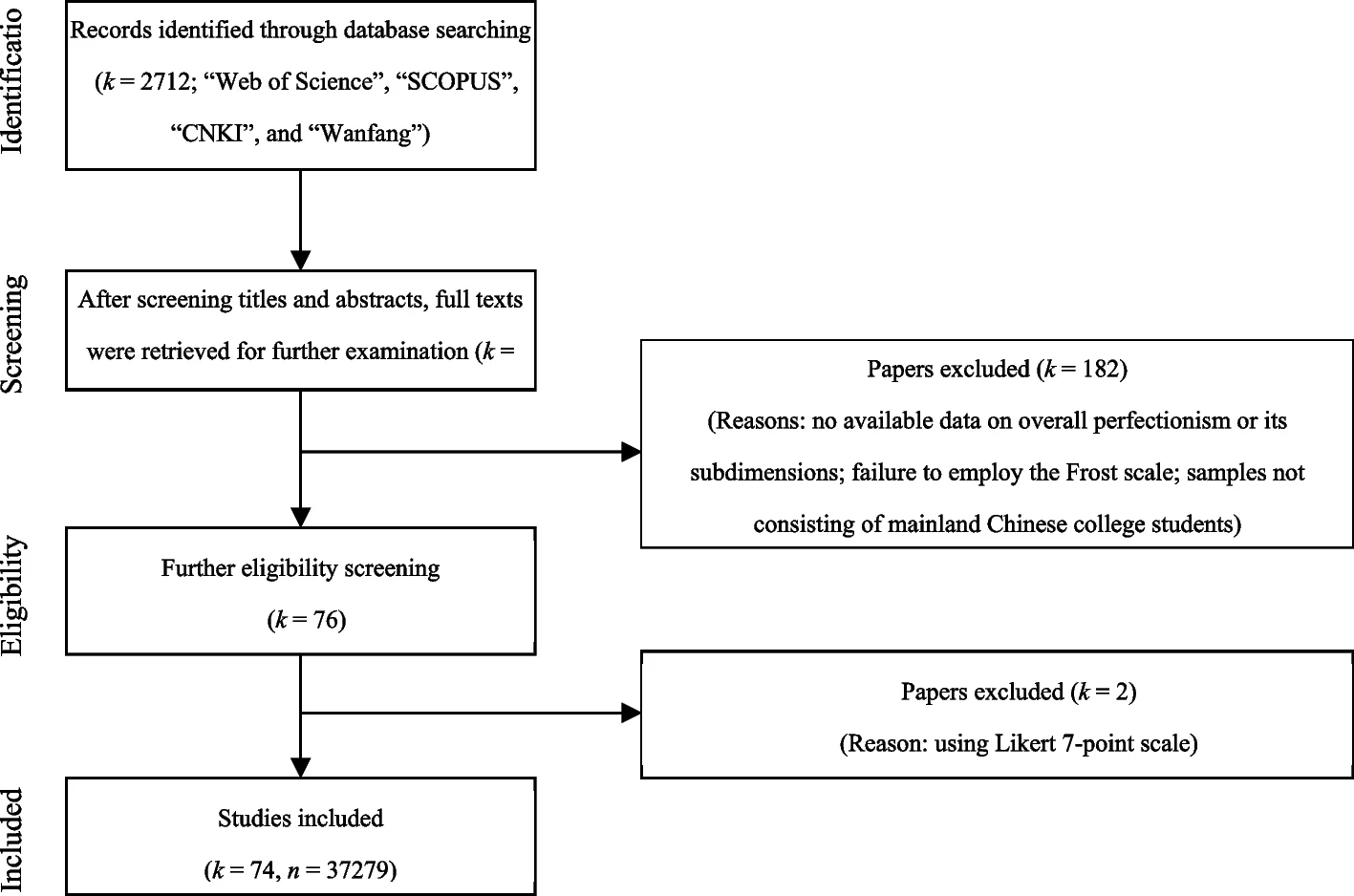
PRISMA flow diagram illustrating the literature selection process for the meta-analysis

On this basis, a total of 2712 studies were initially identified. Titles and abstracts were screened to remove clearly irrelevant records, after which 258 full-text articles were retrieved for further evaluation. Following a detailed review, 74 studies met all criteria and were included in the final analysis, comprising 70 Chinese-language and 4 English-language studies. The total sample size was 37,279, contributing 355 independent means. The study selection process is illustrated in Fig. 1 (PRISMA flow diagram).

### Coding procedure

The coding procedure was conducted independently by two researchers. Data collection years were recorded directly when reported. When unavailable, we followed conventional estimation rules used in prior cross-temporal meta-analytic research: for journal articles, the publication year minus two years was used; for master’s theses, the defense year minus one year was used (e.g., [5, 9, 43, 47]. Perfectionism scores were coded as scale means (*M*) and standard deviations (*SD*),when total scores were reported, these values were divided by the number of items to derive *M* and *SD* [9]. Additional coding included author names, sample size, and male participant counts. Discrepancies were resolved through third-researcher adjudication until consensus was achieved. Complete coding protocols are available in the Supplementary Materials.

### Inclusion of modernization indicators

Data for four modernization indicators in this study were obtained from the China Statistical Yearbook via the CEIDATA platform, a subscription-based service for institutional users. Time-series data were downloaded and compiled for analysis. For each sample, modernization indicators were matched to the corresponding year coded for perfectionism scores. Specifically, when the data collection year was reported, socioeconomic indicators from that same year were used; when the data collection year was unavailable, indicators were matched to the estimated data collection year based on the coding rules described above.

### Analysis

The first research objective examined temporal trajectories of perfectionism and its respective dimensions among Chinese mainland college students. Following prior studies [8, 9], we conducted a meta-regression using a random-effects model. Analyses were performed using the metafor package in R [44]. Year of data collection was entered as the predictor, and inverse-variance weighted scale means served as the outcome variable [27]. Separate analyses were performed for global perfectionism and each dimension. Descriptive statistics are presented in Table 1.

**Table 1:** Descriptive statistics of overall perfectionism and its subdimensions

Variables	*k*	*N*	*M*	*M Range*	*SD*	*SD Range*
Perfectionism	74	37,279	2.95	2.15–3.96	0.58	0.16–0.96
Concern over mistakes	54	23,697	2.27	1.27–3.01	0.78	0.41–1.15
Parental criticism	12	4509	2.14	1.49–2.64	0.65	0.29–0.81
Parental expectations	53	23,508	2.91	1.86–3.74	0.82	0.40–1.04
Personal standards	54	23,697	3.07	2.27–3.78	0.72	0.4–1.08
Organization	54	23,697	3.83	3.04–4.39	0.69	0.37–1.04
Doubts about actions	54	23,697	3.06	2.01–4.62	0.79	0.31–1.34

*k* indicates the number of studies reporting the variable; *N* indicates the total sample size; *M* represents the mean score of the variable; *SD* denotes the standard deviation; M and SD are based on mean-item scores

Furthermore, while Curran and Hill's [8] meta-analysis controlled for gender and nationality, the present study did not control for nationality given the exclusively Chinese sample. Gender composition was controlled in the meta-regression analyses [8]. Results are presented in two sequential models: initial findings on temporal trends followed by analyses incorporating the control variable. Complete meta-regression outputs are reported in Table 2.

**Table 2:** Meta-Regression results

Variables	Model		estimate	*se*	*z*	*p*	*tau*^*2*^	*I*^*2*^	*R*^*2*^	*Q(df)*
Perfectionism	1	Time	**0.016**	0.007	2.39	0.017	9.86%	99.47%	6.11%	19,761.66(72)
	2	Time	**0.018**	0.007	2.52	0.012	9.89%	99.47%	5.81%	19,759.81(71)
		Gender	0.215	0.245	0.88	0.380				
Concern over mistakes	3	Time	**0.015**	0.008	1.73	0.083	10.01%	98.75%	3.57%	4016.25(52)
	4	Time	**0.018**	0.009	2.10	0.035	9.63%	98.70%	7.29%	3970.09(51)
		Gender	**0.456**	0.258	1.77	0.078				
Parental criticism	5	Time	0.018	0.043	0.42	0.674	20.36%	99.50%	0.00%	2230.28(10)
	6	Time	0.017	0.049	0.34	0.737	22.62%	99.56%	0.00%	2230.16(9)
		Gender	−0.049	0.717	−0.07	0.946				
Parental expectations	7	Time	0.004	0.009	0.50	0.615	10.55%	98.70%	0.00%	4025.73(51)
	8	Time	0.004	0.009	0.40	0.692	10.73%	98.72%	0.00%	4017.76(50)
		Gender	−0.111	0.273	−0.40	0.686				
Personal standards	9	Time	0.005	0.008	0.55	0.581	9.22%	98.87%	0.00%	4474.72(52)
	10	Time	0.007	0.008	0.87	0.385	8.99%	98.84%	1.18%	4252.76(51)
		Gender	0.378	0.249	1.52	0.129				
Organization	11	Time	0.001	0.008	0.18	0.860	8.59%	98.84%	0.00%	3561.15(52)
	12	Time	0.002	0.008	0.28	0.782	8.72%	98.86%	0.00%	3548.15(51)
		Gender	0.122	0.245	0.50	0.619				
Doubts about actions	13	Time	**0.032**	0.009	3.46	0.001	11.99%	98.94%	17.37%	4639.27(52)
	14	Time	**0.031**	0.010	3.25	0.001	12.17%	98.96%	16.13%	4638.83(51)
		Gender	−0.144	0.290	−0.50	0.620				

A significance level of 10% (*p* <.100) was adopted for the meta-regression analyses [25]. Estimate represents the unstandardized meta-regression coefficient. Dependent variables were expressed as mean-item scores. Bolded values indicate statistically significant point estimates

The second research objective examined the predictive effects of four modernization indicators on perfectionism and its dimensions. The pairwise correlations among these indicators were reported in Appendix 7. Several indicators were highly correlated, suggesting potential multicollinearity and indicating that the individual coefficients should be interpreted cautiously. Therefore, we standardized and combined these variables into a composite modernization index to assess their overall predictive power. We used standardized averaging rather than PCA because our goal was to create a theoretically interpretable index of broad societal modernization, while giving each theoretically relevant indicator equal weight. Given the high intercorrelations among the indicators, this composite provides a transparent summary of the shared modernization trend.

In the previous section, we identified significant temporal increases in overall perfectionism, concern over mistakes, and doubts about actions. Here, we aim to explain these trends using the modernization indicators. Following prior studies [5, 46], we tested the predictive power of concurrent, 1-year lagged, 3-year lagged, and 5-year lagged modernization indicators.

We conducted weighted regression analyses using SPSS, with weights defined as *ω* = *n*/(*SD*)^2^ [45]. The significance level for this analysis was set at *p* < 0.050, distinct from the *p* < 0.100 threshold used in the meta-regressions. Results are presented in Table 3. Each modernization indicator was examined in a separate weighted regression model rather than entered simultaneously with the other indicators. This approach was used because the modernization indicators were highly intercorrelated, and separate models allowed us to evaluate the bivariate predictive pattern of each indicator while avoiding unstable estimates due to multicollinearity. Because these analyses focused on historical associations between cohort mean scores and macro-level modernization indicators, rather than pooled bivariate effect sizes, conventional publication-bias diagnostics (e.g., funnel plots and Egger’s tests) were considered less informative for the present analyses and therefore were not applied.

**Table 3:** Predicting perfectionism from modernization indicators

		Perfectionism			Concern over mistakes			Doubts about actions		
		β	t	p	β	t	p	β	t	p
Concurrent	GDP per capita	0.102	0.87	0.385	0.202	1.49	0.143	**0.387**	**3.028**	**0.004**
	Urbanization	0.143	1.23	0.225	0.146	1.07	0.291	**0.351**	**2.703**	**0.009**
	Divorce rates	**0.33**	**2.97**	**0.004**	−0.083	−0.60	0.549	0.193	1.416	0.163
	Household size	0.065	0.55	0.584	−0.202	−1.49	0.143	**−0.302**	**−2.285**	**0.026**
	Overall modernization	**0.313**	**2.798**	**0.007**	−0.001	0.00	0.997	0.26	1.938	0.058
1-Year lagged	GDP per capita	0.12	1.023	0.31	0.189	1.385	0.172	**0.395**	**3.097**	**0.003**
	Urbanization	0.136	1.17	0.247	0.15	1.088	0.282	**0.361**	**2.789**	**0.007**
	Divorce rates	0.145	1.24	0.218	0.01	0.1	0.921	0.229	1.693	0.097
	Household size	0.057	0.49	0.628	−0.215	−1.586	0.119	**−0.296**	**−2.235**	**0.03**
	Overall modernization	0.227	1.98	0.052	0.028	0.203	0.84	**0.291**	**2.191**	**0.033**
3-Year lagged	GDP per capita	0.103	0.88	0.385	0.211	1.554	0.126	**0.405**	**3.197**	**0.002**
	Urbanization	0.126	1.08	0.286	0.155	1.133	0.262	**0.359**	**2.774**	**0.008**
	Divorce rates	0.136	1.16	0.249	0.187	1.375	0.175	**0.356**	**2.749**	**0.008**
	Household size	**−0.249**	**−2.18**	**0.032**	0.024	0.176	0.861	**−0.292**	**−2.204**	**0.032**
	Overall modernization	0.109	0.94	0.353	0.208	1.531	0.132	**0.358**	**2.764**	**0.008**
5-Year lagged	GDP per capita	0.102	0.87	0.387	0.234	1.733	0.089	**0.419**	**3.328**	**0.002**
	Urbanization	0.125	1.07	0.289	0.162	1.185	0.241	**0.359**	**2.773**	**0.008**
	Divorce rates	0.095	0.81	0.421	0.204	1.499	0.14	**0.402**	**3.17**	**0.003**
	Household size	−0.227	−1.98	0.051	−0.089	−0.643	0.523	−0.218	−1.609	0.114
	Overall modernization	0.094	0.804	0.424	0.191	1.404	0.166	**0.414**	**3.282**	**0.002**

*β* represents the standardized regression coefficient. Dependent variables are expressed as mean-item scores. Bolded values indicate statistically significant results

## Results

### Temporal trends in college students’ perfectionism

**Fig. 2:**
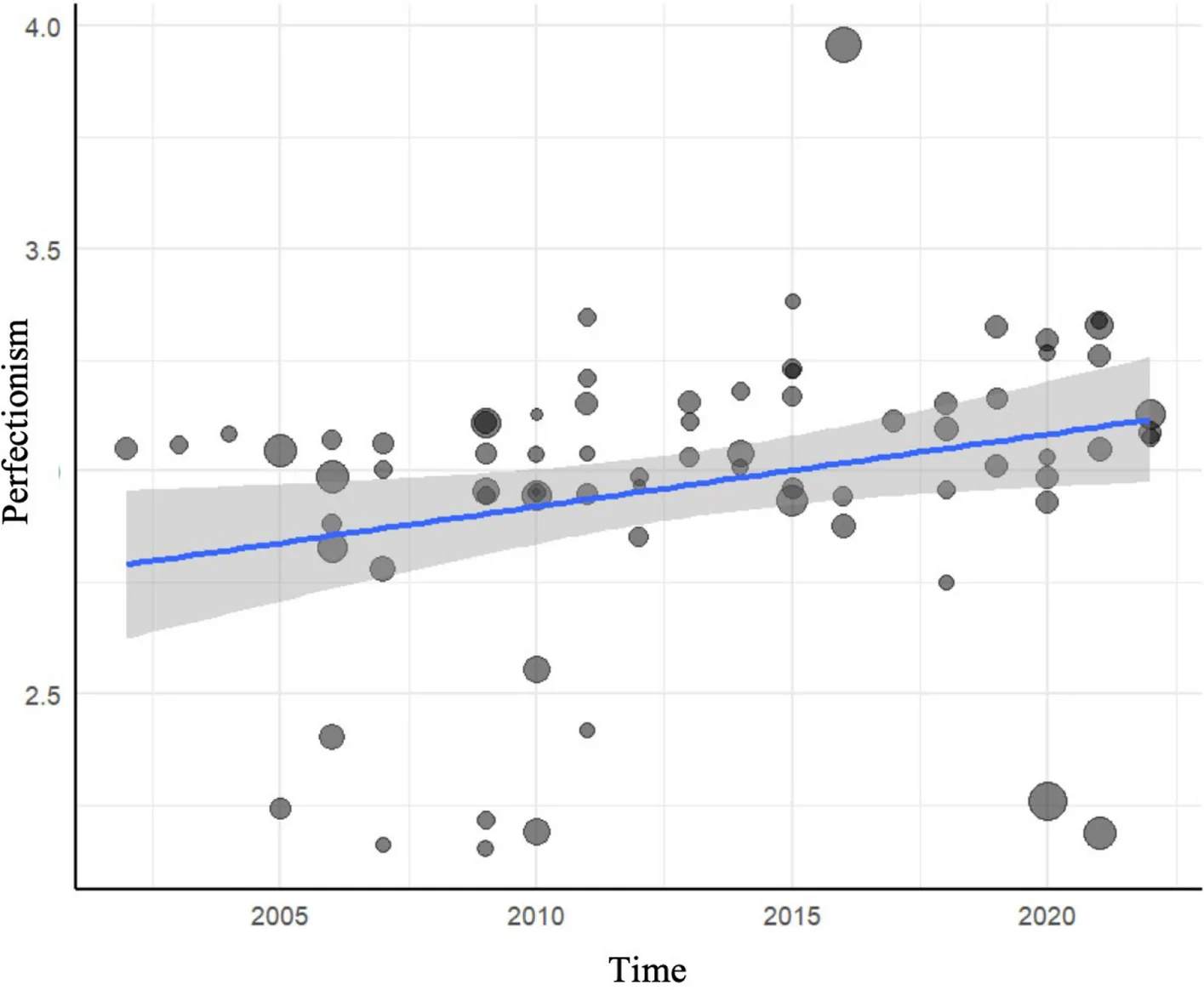
Changes in perfectionism among mainland Chinese college students (2002–2022)

Overall perfectionism and two subdimensions—concern over mistakes and doubts about actions—showed significant temporal effects. Following prior meta-regression conventions [25], significance was determined at *p* < 0.100. It is also consistent with meta-analytic guidance in which *p* < 0.100 is sometimes treated as trend-level evidence [15], Chapter 8). This decision was motivated by the relatively limited statistical power often associated with meta-regression analyses involving a modest number of studies. Exact *p*-values are reported throughout to allow readers to apply their preferred significance threshold. As shown in Table 2, for overall perfectionism, the year predictor yielded a point estimate of 0.016 (*p* = 0.017) in Model 1 and 0.018 (*p* = 0.012) in Model 2, indicating a significant upward trend among mainland Chinese college students over time. To visualize this pattern, we used the *ggplot2* package in R to plot the time series of mean perfectionism scores (see Fig. 2). The distribution of data points and the fitted trend line clearly illustrate a rising trajectory across the study period.

**Fig. 3:**
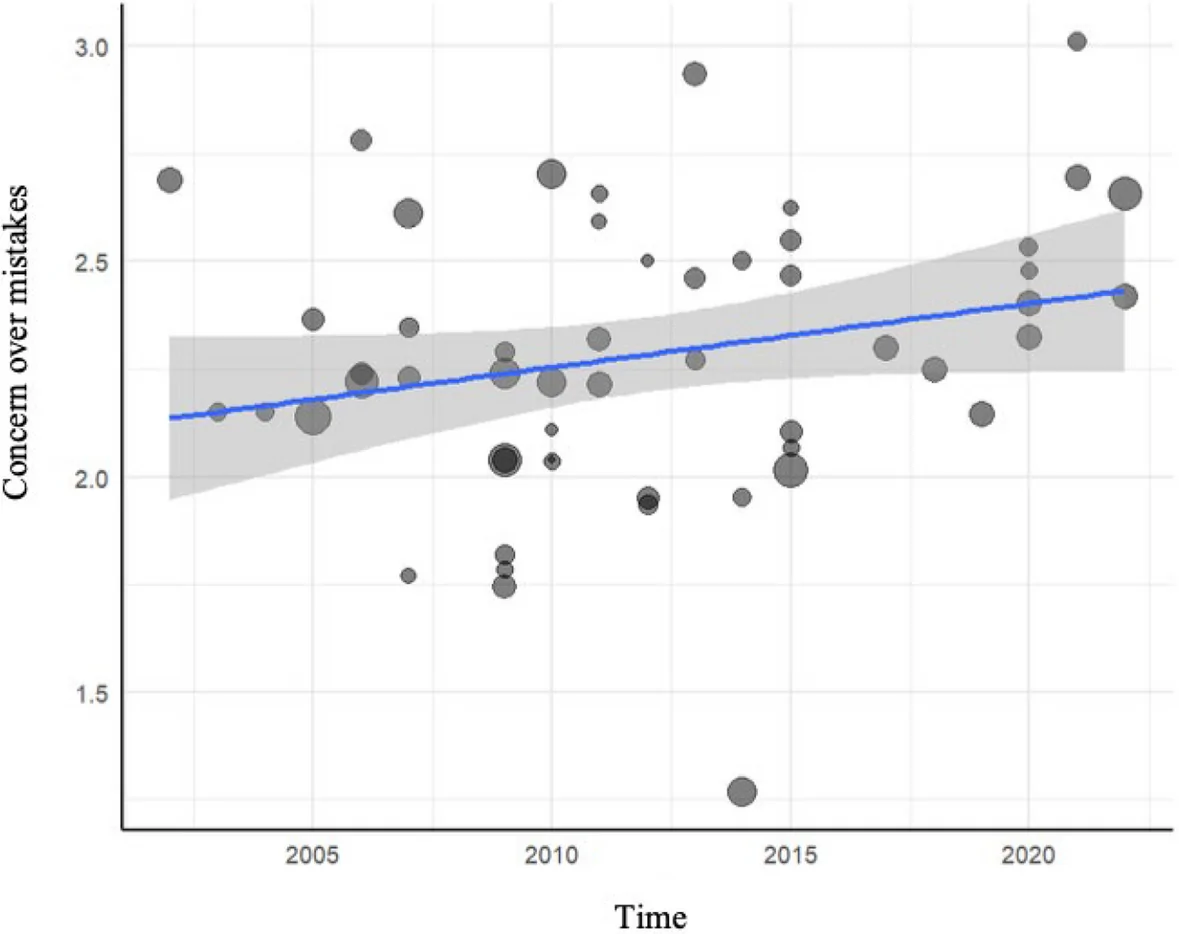
Changes in concern over mistakes among mainland Chinese college students (2002–2022)

Results also indicate an increase in concern over mistakes among mainland Chinese college students over time. Model 3 showed a year coefficient of 0.015 (*p* = 0.083 < 0.100); Model 4 included year (0.018, *p* = 0.035 < 0.100) and gender (0.456, *p* = 0.078 < 0.100)^1^ coefficients. Gender, coded as the proportion of males, positively predicted this dimension, suggesting that higher male ratios correspond with higher scores. Figure 3 depicts this upward trend clearly through sample distributions and fitted lines.

**Fig. 4:**
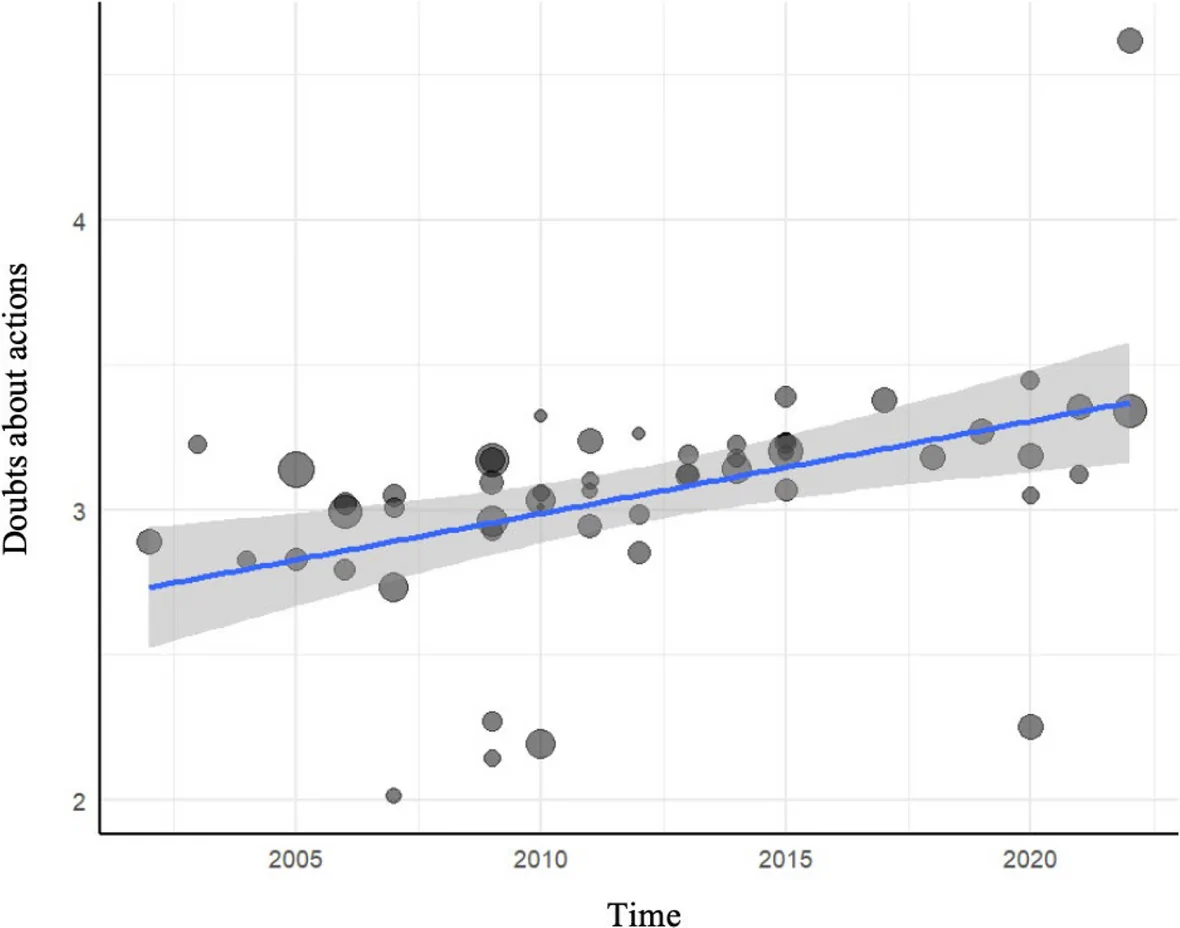
Changes in doubts about actions among mainland Chinese college students (2002–2022)

Models 13 and 14 yielded year coefficients of 0.032 (*p* = 0.001 < 0.100) and 0.031 (*p* = 0.001 < 0.100), respectively, indicating a significant upward trend. This suggests that doubts about actions among mainland Chinese college students have increased over time. Figure 4 illustrates this rising trend clearly.

Other dimensions of perfectionism in this study—parental criticism, parental expectations, personal standards, and organization—showed no significant change over time. For instance, parental criticism yielded year effects with p-values of 0.674 and 0.737 in Models 5 and 6, respectively, both above the significance threshold (*p* > 0.100).

Finally, the standardized mean difference (d) was computed to quantify the magnitude of temporal changes in perfectionism and its dimensions, following prior meta-analytic procedures [9, 5].

### Calculation of effect sizes (Cohen’s d) over time

This section aims to assess the degree of change in perfectionism over time, providing a clearer understanding of how substantially it has shifted between 2002 and 2022. *First*, we estimated the change in mean scores by inputting the years 2002 and 2022 into the meta-regression equations and calculating the difference, following the procedure outlined by Curran & Hill [8]. We used Models 1, 3, and 13, which excluded gender as a covariate. *Second*, we calculated the weighted average standard deviation (*SD*). *Third*, Cohen’s d was computed by dividing the mean change by the weighted *SD*. Specifically, for perfectionism, the regression equation was: y = 0.016x—29.777, yielding d = ((0.016 * 2022)—(0.016 * 2002))/0.579 = 0.55. For concern over mistakes, the regression equation was: y = 0.015x—26.174, yielding d = ((0.015 * 2022)—(0.015 * 2002))/0.782 = 0.38. For doubts about actions, y = 0.032x − 64.185, yielding d = ((0.032 × 2022) − (0.032 × 2002))/0.79 = 0.81.

Per Cohen's [7] benchmarks (d ≈ 0.2/0.5/0.8 indicating small/medium/large effects), Chinese mainland college students exhibited moderate increases in overall perfectionism from 2002 to 2022. Specifically, concern over mistakes showed a small-to-medium increase, whereas doubts about actions demonstrated large changes.

Notably, potential outliers were observed in the bubble plots (Figs. 2, 3 and 4). For instance, one data point in Fig. 2 deviated notably from the rest, slightly shifting the regression intercept when excluded. Following Schmidt and Hunter [35], since the study met all inclusion criteria and showed no methodological anomalies, the outlier was retained. Likewise, outliers in Figs. 3 and 4 were kept, as they likely reflect true heterogeneity rather than design flaws.

### Predictive role of modernization indicators

First, as shown in Table 3, for overall perfectionism, results indicated that the concurrent divorce rate positively predicted perfectionism. The concurrent overall modernization also showed a positive association with perfectionism (*β* = 0.313, *p* = 0.007 < 0.010). The 3-year lagged household size negatively predicted perfectionism (*β* = −0.249, *p* = 0.032 < 0.050).

Second, regarding doubts about actions, we found that same-year GDP per capita (*β* = 0.387, *p* = 0.004 < 0.010) and urbanization rate (*β* = 0.351, *p* = 0.009 < 0.010) significantly positively predicted this dimension, while household size negatively predicted it (*β* = − 0.302, *p* = 0.026 < 0.050). The 1-year lagged GDP per capita (*β* = 0.395, *p* = 0.003 < 0.010), urbanization rate (*β* = 0.361, *p* = 0.007 < 0.010), and overall modernization index (*β* = 0.291, *p* = 0.033 < 0.050) also positively predicted doubts about actions, whereas household size showed a negative association (*β* = −0.296, *p* = 0.030 < 0.050). Similarly, the 3-year lagged GDP per capita (*β* = 0.405, *p* = 0.002 < 0.010), urbanization rate (*β* = 0.359, *p* = 0.008 < 0.010), divorce rate (*β* = 0.356, *p* = 0.008 < 0.010), and modernization index (*β* = 0.358, *p* = 0.008 < 0.010) all positively predicted doubts about actions, while household size showed a negative effect (*β* = − 0.292, *p* = 0.032 < 0.050). For the 5-year lag, GDP per capita (*β* = 0.419, *p* = 0.002 < 0.010), urbanization rate (*β* = 0.359, *p* = 0.008 < 0.010), divorce rate (*β* = 0.402, *p* = 0.003 < 0.010), and modernization index (*β* = 0.414, *p* = 0.002 < 0.010) remained significant positive predictors, with household size consistently showing a negative association (*β* = − 0.218, *p* = 0.114 > 0.010). In contrast, for the concern over mistakes, none of the four modernization indicators exhibited statistically significant predictive effects across all time lags.

In recent years, the Granger causality test has been applied in psychological research on social change. For example, Cai et al. [4] found that GDP per capita could significantly predict subjective well-being 1, 2, and 4 years later. To further examine the impact of modernization on college students’ perfectionism, we conducted Granger causality tests for each of the four modernization indicators. Before testing, we used the Augmented Dickey-Fuller (ADF) test to assess the stationarity of the data. Results showed that all series became stationary after first-order differencing (*p* < 0.010). Granger causality tests were then performed using EViews (see Table 4).

**Table 4:** Results of Granger causality tests

	**lag length**	**GDP per capita**	**Urbanization**	**Divorce rates**	**Household size**	**Overall modernization**
Perfectionism	1 Year	*F* = 3.10895(*p* = 0.0958)	*F* = 2.69343(*p* = 0.1191)	*F* = 1.37935(*p* = 0.2564)	*F* = 1.08594(*p* = 0.3120)	*F* = 2.61013(*p* = 0.1246)
	2 Year	*F* = 2.32117(*p* = 0.1347)	*F* = 2.10346(*p* = 0.1589)	*F* = 1.95422(*p* = 0.1784)	*F* = 3.76177^*^(*p* = 0.0493)	*F* = 2.64001(*p* = 0.1064)
	3 Year	*F* = 3.82367^*^(*p* = 0.0424)	*F* = 3.06562(*p* = 0.0731)	*F* = 3.24295(*p* = 0.0641)	*F* = 6.97783(*p* = 0.0068)	*F* = 6.75370^*^(*p* = 0.0076)
	4 Year	*F* = 0.48405(*p* = 0.7478)	*F* = 0.49891(*p* = 0.7380)	*F* = 0.67034(*p* = 0.6306)	*F* = 1.63902(*p* = 0.2556)	*F* = 2.54780(*p* = 0.1212)
	5 Year	*F* = 0.24543(*p* = 0.9253)	*F* = 0.40325(*p* = 0.8292)	*F* = 0.74897(*p* = 0.6206)	*F* = 0.82115(*p* = 0.5830)	*F* = 1.97319(*p* = 0.2368)
Concern over mistakes	1 Year	*F* = 2.61813(*p* = 0.1241)	*F* = 3.21327(*p* = 0.0909)	*F* = 1.51220(*p* = 0.2356)	*F* = 8.85033^*^(*p* = 0.0085)	*F* = 1.19591(*p* = 0.2894)
	2 Year	*F* = 1.38642(*p* = 0.2823)	*F* = 1.35478(*p* = 0.2898)	*F* = 2.69579(*p* = 0.1022)	*F* = 3.85796^*^(*p* = 0.0463)	*F* = 6.40373^*^(*p* = 0.0106)
	3 Year	*F* = 1.30957(*p* = 0.3204)	*F* = 1.64388(*p* = 0.2360)	*F* = 1.84778(*p* = 0.1969)	*F* = 3.10677(*p* = 0.0709)	*F* = 4.80519^*^(*p* = 0.0224)
	4 Year	*F* = 0.76227(*p* = 0.5782)	*F* = 1.74325(*p* = 0.2333)	*F* = 1.63426(*p* = 0.2567)	*F* = 3.21748(*p* = 0.0749)	*F* = 5.19982^*^(*p* = 0.0232)
	5 Year	*F* = 1.42032(*p* = 0.3548)	*F* = 1.46561(*p* = 0.3425)	*F* = 1.37243(*p* = 0.3684)	*F* = 4.33782(*p* = 0.0666)	*F* = 4.34175(*p* = 0.0665)
Doubts about actions	1 Year	*F* = 4.02816(*p* = 0.0609)	*F* = 4.31474(*p* = 0.0533)	*F* = 0.47319(*p* = 0.5008)	*F* = 3.34255(*p* = 0.0851)	*F* = 1.56625(*p* = 0.2277)
	2 Year	*F* = 4.29065^*^(*p* = 0.0352)	*F* = 4.78470^*^(*p* = 0.0261)	*F* = 8.24862^*^(*p* = 0.0043)	*F* = 6.83786^*^(*p* = 0.0085)	*F* = 1.65305(*p* = 0.2267)
	3 Year	*F* = 3.53683(*p* = 0.0518)	*F* = 1.57413(*p* = 0.2513)	*F* = 3.61342^*^(*p* = 0.0491)	*F* = 2.48269(*p* = 0.1153)	*F* = 1.40446(*p* = 0.2934)
	4 Year	*F* = 4.46418^*^(*p* = 0.0345)	*F* = 4.93379^*^(*p* = 0.0266)	*F* = 8.61262^*^(*p* = 0.0054)	*F* = 7.02890^*^(*p* = 0.0099)	*F* = 2.75543(*p* = 0.1038)
	5 Year	*F* = 2.75435(*p* = 0.1452)	*F* = 2.38731(*p* = 0.1808)	*F* = 2.66950(*p* = 0.1525)	*F* = 6.33460^*^(*p* = 0.0320)	*F* = 1.75229(*p* = 0.2766)

Perfectionism, concern over mistakes, and doubts about actions were expressed as mean-item scores

The overall modernization significantly predicted college students’ perfectionism three years later (*p* = 0.008), as well as concern over mistakes two (*p* = 0.011), three (*p* = 0.022), and four years (*p* = 0.023) later. Among the individual indicators, GDP per capita predicted perfectionism after a three-year lag (*p* = 0.042), while household size predicted it after two years (*p* = 0.049). Household size also predicted concern over mistakes one (*p* = 0.009) and two years (*p* = 0.046) later. Regarding doubts about actions, GDP per capita showed a significant positive prediction at a two-year lag (*p* = 0.035); urbanization rate predicted it after two (*p* = 0.026) and four years (*p* = 0.027); divorce rate predicted it after two (*p* = 0.004) and three years (*p* = 0.049); and household size negatively predicted it after two (*p* = 0.009), four (*p* = 0.009), and five years (*p* = 0.032). Although Granger causality does not imply true causal relationships, these results provide important statistical evidence that China’s modernization—both overall and in specific domains—may predict subsequent changes in perfectionism and its dimensions over time.

### Robustness check

As a robustness check, we examined the three significant meta-regression results by identifying potential influential cases using the outlier detection methods recommended in the *metafor* package. We then re-conducted these meta-regression analyses after removing the identified cases (see Appendices 3–6). The results remained significant, providing additional support for the robustness of the findings. In addition, we conducted a separate sensitivity analysis to examine whether the year-coding procedure affected the temporal trend. Specifically, we re-ran the meta-regression after excluding studies with explicitly reported survey years and retaining only studies whose data collection years were estimated using the imputation rules (see Appendix 8). The temporal trend remained significant, suggesting that the observed increase in perfectionism over time may not have been substantially affected by the year-imputation procedure.

## Discussion

### Rising perfectionism among Chinese college students (2002–2022)

China's modernization has precipitated profound socioeconomic transformations, concurrently reshaping psychological characteristics of successive college student cohorts [3]. This study demonstrates significant upward trajectories in perfectionism (d = 0.55) and its dimensions—concern over mistakes (d = 0.38) and doubts about actions (d = 0.81)—among mainland Chinese college students during 2002–2022. Perfectionism has intensified in the two decades post-WTO accession. College students are increasingly pursuing perfection and setting higher standards for themselves.

Meta-analysis evidence indicates deteriorating mental health indicators among Chinese college students over the past thirty years [6, 49]. Given perfectionism's established negative correlation with mental health and positive association with anxiety [38]—positioned as a potential precursor to these conditions [37]—its rise may partially explain worsening mental health outcomes during this period.

Using cross-temporal meta-analysis and the Hewitt and Flett Multidimensional Perfectionism Scale, Curran and Hill [8] found that from 1989 to 2016, three dimensions of perfectionism—self-oriented, socially prescribed, and other-oriented—showed significant increases among college students in the U.S., U.K., and Canada, with effect sizes (d) of 0.12, 0.40, and 0.19, respectively. Although direct score comparisons are not possible due to different measurements, prior research has shown moderate correlations between Hewitt’s scale and Frost’s scale used in the present study [11]. The observed rise in perfectionism across studies using different instruments may suggest a cross-national and cross-cultural trend, though further research with more countries is needed to confirm this pattern.

This study observed several increased trends across perfectionism dimensions: concern over mistakes (d = 0.38) and doubts about actions (d = 0.81) increased significantly. Parental criticism, parental expectations, personal standards, and organization remained stable. These findings underscore the multifaceted nature of perfectionism [12] and suggest that its dimensions may be differentially sensitive to broader socio-cultural transformations.

Both concern over mistakes and doubts about actions belong to the "negative-oriented" dimensions of perfectionism, which are strongly associated with maladaptive psychological outcomes such as anxiety, procrastination, and depression [41]. This interpretation is also consistent with Stoeber and Otto’s [41] distinction between perfectionistic strivings and perfectionistic concerns. Concern over mistakes and doubts about actions are conceptually closer to perfectionistic concerns, which are more strongly associated with maladaptive psychological outcomes, whereas dimensions such as personal standards and organization are closer to perfectionistic strivings. The increase in these maladaptive dimensions may be linked to the increasingly competitive academic and social context faced by Chinese college students. Recent research suggests that the academic involution atmosphere in Chinese universities is associated with greater academic pressure and mental exhaustion among students [24]. In such a context, students may become more sensitive to failure and more likely to regard mistakes as costly, which may contribute to the rise in concern over mistakes. In addition to academic pressure, Chinese college students also face increasing uncertainty in educational and career development. Qualitative evidence suggests that employment competition and reduced opportunities in China contribute to youths' career decision-making difficulties [36]. Such environmental uncertainty may make students more hesitant when evaluating their own choices and actions, which may help explain the rise in doubts about actions. These pressures may help explain why the temporal increase was concentrated in concern over mistakes and doubts about actions rather than in more adaptive dimensions.

In contrast, dimensions more closely related to familial socialization processes—such as parental criticism and parental expectations—did not exhibit significant increases over time. This may suggest that parenting beliefs and practices remained relatively stable during the examined period, or that their influence diminishes during the university years as institutional and societal contexts exert a stronger impact. Similarly, the dimensions of personal standards and organization, often considered more adaptive aspects of perfectionism, showed no marked fluctuation. This relative stability may indicate that students’ standards for self-goal setting and behavioral regulation were less affected by broader sociocultural changes during the observed timeframe.

A pertinent issue worthy of further exploration is whether the observed increase in perfectionism among college students is inherently detrimental. This question requires a dialectical perspective. Evolutionary psychology posits that personality traits are not inherently “good” or “bad”; rather, each trait has an evolutionary basis and its formation and development reflect, to some extent, an individual’s adaptation to their environment [2]. From this viewpoint, one's perfectionism may facilitate environmental adaptation. For instance, a meta-analysis by Madigan [28] found that certain dimensions of perfectionism are positively correlated with students’ academic achievement [28]. Over recent decades, societal expectations for academic performance have increased, and various forms of competition have become more prevalent. The self-imposed high standards characteristic of perfectionism [12] may partly enable students to better meet these environmental demands. Nevertheless, while recognizing the adaptive benefits of perfectionism, it is also important to acknowledge its potential adverse effects on mental health.

### Macro-level correlates of changes in college students' perfectionism

#### Modernization and perfectionism

This study, grounded in Greenfield’s [13] theoretical model, identified four key modernization indicators—per capita GDP, urbanization rate, divorce rate, and household size—to examine their predictive effects on the trajectory of perfectionism among Chinese college students. Utilizing both concurrent and lagged data (1-year, 3-year, and 5-year lags),^2^we found a consistent pattern: higher levels of per capita GDP, urbanization, and divorce rate were positively associated with increased levels of perfectionism among Chinese college students, while smaller household size was negatively associated with perfectionism, suggesting that as household size declines, perfectionism increases. A composite modernization index, calculated as the standardized average of the four indicators, also showed a positive association with overall perfectionism.

These findings are in line with prior theoretical and empirical work. Inglehart and Baker [22] argued that modernization, with economic growth and industrialization, increases per capita GDP and accelerates urban migration, thereby raising urbanization levels. In the Chinese context, decades of rapid economic development have been associated with an increasingly competitive environment. Greenfield [13] suggested that such socioeconomic conditions may be linked to individualistic values and a stronger emphasis on personal success. In educational and cultural domains, this emphasis may manifest as elevated standards and internal pressures—hallmarks of perfectionistic traits [12]. Urbanization may also provide a context for interpreting rising perfectionism, as higher population density and interpersonal proximity may be associated with greater competition and social comparison [13].

Furthermore, modernization may be associated with shifts in social values and family structures. Economic development and expanded education have fostered greater emphasis on autonomy and self-realization, contributing to rising divorce rates and smaller household sizes. For college students, these changes may reflect broader shifts in family support networks. In such environments, students may become more vulnerable to external pressures, which offers one possible interpretation of the association between modernization and perfectionism. In the Chinese context, the negative association between household size and perfectionism may also be related to the long-term influence of the one-child policy. Smaller households often mean fewer sibling resources and less horizontal family support, while parental attention may be more concentrated on the only child. These changes in family structure may increase students’ pressure to meet high standards and avoid failure, offering a culturally specific explanation for the association between smaller household size and higher perfectionism. The present study did not directly measure only-child status or perceived family support; future research could examine these factors to further clarify the family-level mechanisms underlying changes in perfectionism.

Rising levels of GDP per capita, urbanization, and divorce rates, alongside declining household sizes, reflect salient features of China's modernization process. When integrated into a composite modernization index, these indicators collectively predicted increases in perfectionism among Chinese college students. Supplementary evidence from Granger causality tests further supports this relationship, indicating that the composite modernization index significantly predicts student perfectionism. Specifically, GDP per capita and household size emerged as Granger causes of overall perfectionism; household size was a Granger cause of concern over mistakes; and GDP per capita, urbanization rate, divorce rate, and household size were all Granger causes of doubts about actions. Taken together, these findings suggest that the ongoing modernization of Chinese society may be associated with the observed rise in perfectionism among college students.

Although regression analyses showed no significant direct associations between modernization indicators and concern over mistakes, the Granger causality tests suggested that household size and overall modernization improved the prediction of subsequent concern over mistakes beyond its own temporal history. This difference may arise because the regression analyses estimated direct associations, whereas the Granger causality tests examined temporal predictive effects.

#### Individualism and perfectionism

To interpret the macro-level associations observed in this study, Greenfield’s [13] multilevel model provides a useful theoretical perspective. This model posits that socioeconomic change may shape individual development, including psychological traits such as perfectionism. As discussed earlier, modernization may be linked to the rise of perfectionism at the macro level. Beyond this direct pathway, the model also suggests that socioeconomic shifts may shape individual traits indirectly through cultural transformation.

A growing body of research indicates a steady rise in individualistic values in China since the Reform and Opening-up period [3]. Following Greenfield’s model, this cultural shift may provide one theoretical context for interpreting the observed association between modernization and perfectionism. In highly individualistic societies, cultural norms emphasize autonomy, personal goals, self-realization, and individual achievement [14]. These values often place pressure on individuals to strive for high levels of personal success. Over time, such culturally embedded expectations may lead individuals to set unrealistically high standards for themselves, thereby fostering the development of perfectionistic tendencies.

Although individualism is commonly assessed through self-report instruments such as Hofstede’s cultural dimensions questionnaire [20], the lack of continuous longitudinal data in China precludes us from directly examining its predictive role in perfectionism trends. Therefore, the individualism pathway was not directly tested in the present study. Future research could measure cultural values to examine whether changes in individualism help explain the association between modernization and perfectionism more rigorously.

### Theoretical implications

First, this study may represent the first systematic investigation into temporal changes in perfectionism among college students in mainland China, offering valuable empirical evidence for understanding the evolution of this psychological trait in the context of modernization. While longitudinal studies are typically required to track personality trait changes over time, such studies are often constrained by high costs and substantial participant attrition, resulting in a scarcity of longitudinal data for many psychological variables. This limitation hampers a deeper understanding of the developmental trajectory of such traits. Cross-temporal meta-analysis serves as a valuable alternative by leveraging historical data to identify shifts in psychological constructs across time [21].

By applying this method to the study of perfectionism in Chinese college students, the current research contributes to the theoretical literature on personality development. It also provides empirical insights into how Chinese college student cohorts psychologically adapt to rapid social change. More broadly, the findings may offer indirect evidence for psychological transformations that have accompanied China’s reform and opening-up era, thereby expanding the theoretical understanding of personality traits under conditions of societal transition.

Second, this study contributes to the understanding of the antecedents of perfectionism by uncovering the relationship between modernization and the development of perfectionistic traits. Previous research has primarily focused on genetic influences [23] and familial environmental factors [39], with little attention paid to the role of broader societal forces such as modernization. However, modernization often entails technological advancement and structural transformation, producing complex socio-economic and cultural effects that exert profound influence on individual psychological functioning. As such, the topic addressed in this study is both timely and significant.

Macro-level socio-economic variables play a crucial role in shaping and transforming the psychological characteristics of populations and should not be overlooked [13]. In this context, the present study identifies and empirically examines several key indicators of modernization and demonstrates their predictive value in relation to changes in perfectionism among Chinese college students. By adopting a macro-sociological perspective, the study advances our theoretical understanding of perfectionism and highlights how personality traits may be linked to broader societal transformations in the context of China’s modernization. The implications of these findings suggest that future research on the origins of perfectionism should incorporate socio-economic and cultural variables, particularly those associated with modernization. Given that many countries across the globe are currently undergoing similar processes of modernization, the insights provided by this study may offer theoretical relevance beyond the Chinese context, contributing to a more comprehensive and globally applicable understanding of perfectionism.

### Practical implications

First, this study contributes to a clearer public understanding of the rising trend of perfectionism among Chinese college students, drawing attention to a psychological shift that may otherwise be overlooked. While perfectionistic tendencies among college students may be noted anecdotally, this research provides empirical evidence that such tendencies represent a broader trend across student cohorts rather than isolated cases. This trend should be interpreted within the broader context of China’s rapid social and psychological transformation over the past decades. Although perfectionism aligns with long-standing cultural values—such as academic excellence and parental expectations—the present findings suggest that modernization may be an important context for understanding its intensification. By highlighting sociocultural and structural contexts related to this trend, the study offers valuable insights for families, educators, and employers seeking to understand the changing psychological profiles of contemporary college student cohorts. It may also help individuals with high perfectionistic tendencies better contextualize their experiences and foster more effective support or intervention strategies.

Second, this study offers a foundation for psychological interventions and related mental health services. Given that perfectionism is a well-documented risk factor for various psychological issues such as anxiety, depression, and stress [37], identifying its increasing trend provides valuable context for educational administrators and mental health professionals in understanding students’ psychological burdens. Meta-analytic evidence suggests that targeted interventions can effectively reduce perfectionism and associated anxiety [42]. By documenting the rise in specific dimensions of perfectionism among Chinese college students, this study can inform the development of more tailored prevention and intervention programs, helping practitioners design support services that better address students’ evolving mental health needs.

### Limitations and future directions

First, as a cross-temporal meta-analysis, this study was unable to employ random sampling of participants [43], which may limit the representativeness of the included samples. Furthermore, this methodological approach cannot capture intra-individual developmental changes in perfectionism over time, such as how perfectionistic traits evolve with age within the same individual. Future research should consider employing longitudinal designs to address issues related to sample representativeness and to more directly examine within-person changes in perfectionism. In addition, although focusing on college students reduces age heterogeneity across samples, the cross-temporal meta-analytic design cannot formally disentangle age, period, and cohort effects.

Second, while this study explored the predictive role of modernization indicators on perfectionism, it did not incorporate more proximal factors such as cultural values and academic environments, which are likely to exert stronger explanatory power in shaping perfectionistic tendencies. One challenge in operationalizing these constructs lies in the limited availability of longitudinal and continuous measurements over time. For example, capturing cultural change would require repeated administration of validated instruments across multiple time points, which is currently rare in the literature. Future research may consider identifying suitable proxy indicators to indirectly measure these influential variables. In addition, the present study used national-level modernization indicators and did not control for regional economic differences. Given the substantial variation in economic development, educational resources, and employment pressure across provinces and cities in China, future research should examine whether regional socioeconomic conditions moderate temporal changes in perfectionism among college students.

Third, the present study relied on the FMPS to assess perfectionism. However, perfectionism is a multifaceted construct with various conceptualizations and measurement tools. While existing studies using alternative measures remain limited, future meta-analytic work could build upon a broader range of assessments once more empirical evidence accumulates, thereby enhancing the robustness and generalizability of findings on perfectionism trends.

Fourth, although gender composition was controlled by coding the proportion of males in each sample, this study-level indicator only reflects between-study variation in sample composition and cannot capture within-sample gender differences in perfectionism trajectories over time. Future research should further examine whether temporal trends in perfectionism differ across gender groups.

## Conclusion

This cross-temporal meta-analysis integrated 74 studies comprising 355 independent samples (*N* = 37,279) to assess FMPS-assessed perfectionism trajectories among Chinese mainland college students (2002–2022). Key findings revealed: (1) significant increases in overall perfectionism (d = 0.55), concern over mistakes (d = 0.38), and doubts about actions (d = 0.81); (2) positive associations between divorce rate, GDP per capita, urbanization rate, and perfectionism, respectively, contrasted with a negative association between household size and perfectionism; the composite modernization index also showed a positive association with FMPS-assessed perfectionism. Together, these findings suggest that changes in FMPS-assessed perfectionism among Chinese college students may be linked to broader modernization processes, while the modernization-related results should be interpreted as macro-level temporal associations rather than direct causal evidence. These findings should also be generalized cautiously to perfectionism assessed using other measurement frameworks.

## Supplementary Information

Supplementary Material 1.

## Data Availability

The datasets analyzed in this meta-analysis are derived from previously published empirical studies, all of which are cited and listed in Appendix.No new datasets were generated during this study.

## Footnotes

1 Note. Effects with.050 < *p* <.100 were treated as marginally significant and should be interpreted cautiously. Exact *p*-values are reported to allow readers to apply their preferred significance threshold.

2 The concurrent, 1-year, 3-year, and 5-year lagged analyses were used to examine whether modernization indicators were associated with perfectionism across immediate, short-term, and longer-term time windows. These lag lengths were selected to provide a theoretically meaningful description of temporal associations rather than through formal AIC/BIC-based lag selection procedures.
