# Supplementary material for: Perfectionism increases among Chinese college students: a cross-temporal meta-analysis
Source: BMC Psychol. 2026 Jul 13;14:1448. doi: 10.1186/s40359-026-05169-y (PMC13644279; doi:10.1186/s40359-026-05169-y)
Supplement: Supplementary file 1 [file 40359_2026_5169_MOESM1_ESM.docx]

**Appendix One: Coding Information**

| Authors | Y | year | N | n | Gender Ratio (male) | Variables | M | SD |
| --- | --- | --- | --- | --- | --- | --- | --- | --- |
| Yang, H. F. | 2004 | 2002 | 505 | 276 | 54.65% | Concern over mistakes | 2.690 | 0.770 |
| Zi, F. | 2005 | 2003 | 270 | 270 | 100.00% | Concern over mistakes | 2.150 | 0.731 |
| Tong, Y. H. | 2006 | 2004 | 257 | 126 | 49.03% | Concern over mistakes | 2.149 | 0.783 |
| Zhang, P. | 2006 | 2005 | 1197 | 572 | 47.79% | Concern over mistakes | 2.139 | 0.410 |
| Li, L. | 2006 | 2005 | 392 | 104 | 26.53% | Concern over mistakes | 2.368 | 0.630 |
| Huang, F. | 2007 | 2006 | 362 | 125 | 34.53% | Concern over mistakes | 2.780 | 0.470 |
| Yuqing Song | 2008 | 2006 | 961 | 525 | 54.63% | Concern over mistakes | 2.221 | 0.598 |
| Yang, X. Y. | 2008 | 2006 | 375 | 186 | 49.60% | Concern over mistakes | 2.240 | 0.830 |
| Wang, C. N. | 2008 | 2007 | 189 | 63 | 33.33% | Concern over mistakes | 1.770 | 0.720 |
| Shi, B. | 2009 | 2007 | 700 | 237 | 33.86% | Concern over mistakes | 2.610 | 0.740 |
| Li, R. | 2008 | 2007 | 306 | 152 | 49.67% | Concern over mistakes | 2.346 | 0.847 |
| Yu, S. J. | 2008 | 2007 | 419 | 181 | 43.20% | Concern over mistakes | 2.230 | 0.910 |
| Zhang, L. G. | 2011 | 2009 | 297 | 144 | 48.48% | Concern over mistakes | 1.820 | 0.550 |
| Zhang, L. G. | 2010 | 2009 | 224 | 97 | 43.30% | Concern over mistakes | 1.786 | 0.591 |
| Zhong, Y. H. | 2010 | 2009 | 296 | 206 | 69.59% | Concern over mistakes | 2.290 | 0.630 |
| Ye, Y. S. | 2010 | 2009 | 988 | N/A | 41.10% | Concern over mistakes | 2.040 | 0.740 |
| Sun, J. | 2011 | 2009 | 486 | N/A | 41.10% | Concern over mistakes | 2.040 | 0.740 |
| Wang, L. K. | 2010 | 2009 | 792 | 301 | 38.01% | Concern over mistakes | 2.240 | 0.780 |
| Su, Y. J. | 2010 | 2009 | 426 | 156 | 36.62% | Concern over mistakes | 1.745 | 0.791 |
| Wang, J. | 2012 | 2010 | 127 | 0 | 0.00% | Concern over mistakes | 2.110 | 0.680 |
| Zheng, X. H. | 2011 | 2010 | 755 | 301 | 39.87% | Concern over mistakes | 2.220 | 0.735 |
| Wang, M. M. | 2012 | 2010 | 80 | 27 | 33.75% | Concern over mistakes | 2.040 | 0.835 |
| Kang, H. J. | 2012 | 2010 | 228 | 80 | 35.09% | Concern over mistakes | 2.035 | 0.915 |
| Guo, H. M. | 2012 | 2010 | 761 | N/A | 41.10% | Concern over mistakes | 2.703 | 1.117 |
| Wang, W. J. | 2013 | 2011 | 208 | 86 | 41.35% | Concern over mistakes | 2.660 | 0.590 |
| Jin, Z. | 2012 | 2011 | 174 | 96 | 55.17% | Concern over mistakes | 2.592 | 0.617 |
| Yang, Y. Y. | 2012 | 2011 | 441 | 175 | 39.68% | Concern over mistakes | 2.320 | 0.797 |
| He, Q. | 2012 | 2011 | 500 | 377 | 75.40% | Concern over mistakes | 2.215 | 0.897 |
| Yuan, Y. | 2014 | 2012 | 120 | 17 | 14.17% | Concern over mistakes | 2.500 | 0.590 |
| Tang, H. Y. | 2014 | 2012 | 275 | 69 | 25.09% | Concern over mistakes | 1.934 | 0.729 |
| Chen, H. | 2014 | 2012 | 383 | 205 | 53.52% | Concern over mistakes | 1.950 | 0.800 |
| Li, Y. L. | 2015 | 2013 | 285 | 101 | 35.44% | Concern over mistakes | 2.272 | 0.847 |
| Zheng, S. | 2014 | 2013 | 342 | 138 | 40.35% | Concern over mistakes | 2.462 | 0.878 |
| Peng, C. | 2014 | 2013 | 475 | 252 | 53.05% | Concern over mistakes | 2.934 | 1.146 |
| Wang, Z. H. | 2016 | 2014 | 230 | 74 | 32.17% | Concern over mistakes | 1.950 | 0.700 |
| Zhang, J. Y. | 2017 | 2014 | 266 | 86 | 32.33% | Concern over mistakes | 2.500 | 0.800 |
| Liu, Y. L. | 2016 | 2014 | 771 | 327 | 42.41% | Concern over mistakes | 1.265 | 0.883 |
| Wang, Y. | 2016 | 2015 | 402 | 0 | 0.00% | Concern over mistakes | 2.104 | 0.563 |
| Yu, Z. X. | 2017 | 2015 | 201 | 54 | 26.87% | Concern over mistakes | 2.067 | 0.570 |
| Bian, Z. W. | 2017 | 2015 | 346 | 154 | 44.51% | Concern over mistakes | 2.467 | 0.728 |
| Song, Y. Q. | 2017 | 2015 | 189 | 87 | 46.03% | Concern over mistakes | 2.623 | 0.837 |
| Kuang, L. P. | 2017 | 2015 | 1097 | 373 | 34.00% | Concern over mistakes | 2.013 | 0.903 |
| Zhang, Y. Z. | 2016 | 2015 | 348 | 113 | 32.47% | Concern over mistakes | 2.550 | 1.120 |
| Sun, L. L. | 2018 | 2017 | 502 | 147 | 29.28% | Concern over mistakes | 2.300 | 0.965 |
| Ren, X. M. | 2019 | 2018 | 498 | 193 | 38.76% | Concern over mistakes | 2.250 | 0.962 |
| Li, S. N. | 2020 | 2019 | 500 | 79 | 15.80% | Concern over mistakes | 2.147 | 0.898 |
| Wang, Z. P. | 2021 | 2020 | 505 | 260 | 51.49% | Concern over mistakes | 2.403 | 0.899 |
| Zhang, J. Y. | 2021 | 2020 | 218 | 110 | 50.46% | Concern over mistakes | 2.480 | 0.900 |
| Hao, Y. Q. | 2021 | 2020 | 529 | 120 | 22.68% | Concern over mistakes | 2.325 | 0.901 |
| Yuan, L. | 2022 | 2020 | 249 | 110 | 44.18% | Concern over mistakes | 2.533 | 1.052 |
| Chengjiang Han | 2022 | 2021 | 243 | 203 | 83.54% | Concern over mistakes | 3.010 | 0.930 |
| Yang, S. H. | 2022 | 2021 | 505 | 217 | 42.97% | Concern over mistakes | 2.697 | 0.932 |
| Guo, K. Y. | 2023 | 2022 | 1006 | 401 | 39.86% | Concern over mistakes | 2.660 | 0.770 |
| He, L. T. | 2023 | 2022 | 496 | 116 | 23.39% | Concern over mistakes | 2.418 | 0.828 |
| Jin, Z. | 2012 | 2011 | 174 | 96 | 55.17% | Parental criticism | 2.575 | 0.293 |
| Yuqing Song | 2008 | 2006 | 961 | 525 | 54.63% | Parental criticism | 1.645 | 0.518 |
| Zhang, L. G. | 2010 | 2009 | 224 | 97 | 43.30% | Parental criticism | 1.487 | 0.549 |
| Zhang, L. G. (duplicate) | 2011 | 2009 | 297 | 144 | 48.48% | Parental criticism | 1.570 | 0.560 |
| Wang, W. J. | 2013 | 2011 | 208 | 86 | 41.35% | Parental criticism | 2.580 | 0.600 |
| Zhong, Y. H. | 2010 | 2009 | 296 | 206 | 69.59% | Parental criticism | 2.540 | 0.670 |
| Huang, F. | 2007 | 2006 | 362 | 125 | 34.53% | Parental criticism | 2.520 | 0.680 |
| Zi, F. | 2005 | 2003 | 270 | 270 | 100.00% | Parental criticism | 2.458 | 0.719 |
| Yuan, Y. | 2014 | 2012 | 120 | 17 | 14.17% | Parental criticism | 2.640 | 0.720 |
| Shi, B. | 2009 | 2007 | 700 | 237 | 33.86% | Parental criticism | 2.290 | 0.740 |
| Yang, H. F. | 2004 | 2002 | 505 | 276 | 54.65% | Parental criticism | 2.090 | 0.790 |
| Li, L. | 2006 | 2005 | 392 | 104 | 26.53% | Parental criticism | 2.533 | 0.813 |
| Jin, Z. | 2012 | 2011 | 174 | 96 | 55.17% | Parental expectation | 3.016 | 0.395 |
| Zhang, P. | 2006 | 2005 | 1197 | 572 | 47.79% | Parental expectation | 2.978 | 0.470 |
| Wang, J. | 2012 | 2010 | 127 | 0 | 0.00% | Parental expectation | 3.310 | 0.560 |
| Zheng, X. H. | 2011 | 2010 | 755 | 301 | 39.87% | Parental expectation | 2.250 | 0.590 |
| Huang, F. | 2007 | 2006 | 362 | 125 | 34.53% | Parental expectation | 2.970 | 0.620 |
| He, L. T. | 2023 | 2022 | 496 | 116 | 23.39% | Parental expectation | 3.080 | 0.622 |
| Yu, Z. X. | 2017 | 2015 | 201 | 54 | 26.87% | Parental expectation | 2.986 | 0.650 |
| Zhang, L. G. | 2011 | 2009 | 297 | 144 | 48.48% | Parental expectation | 2.210 | 0.690 |
| Zhang, J. Y. | 2021 | 2020 | 218 | 110 | 50.46% | Parental expectation | 3.120 | 0.690 |
| Zhang, L. G. | 2010 | 2009 | 224 | 97 | 43.30% | Parental expectation | 2.110 | 0.693 |
| Zhong, Y. H. | 2010 | 2009 | 296 | 206 | 69.59% | Parental expectation | 2.850 | 0.700 |
| Wang, W. J. | 2013 | 2011 | 208 | 86 | 41.35% | Parental expectation | 3.030 | 0.700 |
| Wang, Y. | 2016 | 2015 | 402 | 0 | 0.00% | Parental expectation | 2.949 | 0.708 |
| Li, R. | 2008 | 2007 | 306 | 152 | 49.67% | Parental expectation | 2.609 | 0.719 |
| Wang, L. K. | 2010 | 2009 | 792 | 301 | 38.01% | Parental expectation | 2.950 | 0.724 |
| Yang, H. F. | 2004 | 2002 | 505 | 276 | 54.65% | Parental expectation | 3.110 | 0.750 |
| Ye, Y. S. | 2010 | 2009 | 988 | N/A | 41.10% | Parental expectation | 3.130 | 0.760 |
| Sun, J. | 2011 | 2009 | 486 | N/A | 41.10% | Parental expectation | 3.130 | 0.760 |
| Song, Y. Q. | 2017 | 2015 | 189 | 87 | 46.03% | Parental expectation | 3.236 | 0.766 |
| Yuan, Y. | 2014 | 2012 | 120 | 17 | 14.17% | Parental expectation | 2.980 | 0.770 |
| Shi, B. | 2009 | 2007 | 700 | 237 | 33.86% | Parental expectation | 3.250 | 0.770 |
| Yuqing Song | 2008 | 2006 | 961 | 525 | 54.63% | Parental expectation | 2.766 | 0.796 |
| Li, L. | 2006 | 2005 | 392 | 104 | 26.53% | Parental expectation | 2.844 | 0.798 |
| Chengjiang Han | 2022 | 2021 | 243 | 203 | 83.54% | Parental expectation | 3.110 | 0.800 |
| Bian, Z. W. | 2017 | 2015 | 346 | 154 | 44.51% | Parental expectation | 3.160 | 0.806 |
| Yang, Y. Y. | 2012 | 2011 | 441 | 175 | 39.68% | Parental expectation | 3.020 | 0.836 |
| Zi, F. | 2005 | 2003 | 270 | 270 | 100.00% | Parental expectation | 3.018 | 0.845 |
| Peng, C. | 2014 | 2013 | 475 | 252 | 53.05% | Parental expectation | 3.106 | 0.863 |
| Tong, Y.H. | 2006 | 2004 | 257 | 126 | 49.03% | Parental expectation | 3.192 | 0.864 |
| Yang, S.H. | 2022 | 2021 | 505 | 217 | 42.97% | Parental expectation | 3.180 | 0.878 |
| Zhang, J.Y. | 2017 | 2014 | 266 | 86 | 32.33% | Parental expectation | 3.020 | 0.880 |
| Ren, X.M. | 2019 | 2018 | 498 | 193 | 38.76% | Parental expectation | 3.210 | 0.884 |
| Guo, K.Y. | 2023 | 2022 | 1006 | 401 | 39.86% | Parental expectation | 3.180 | 0.890 |
| Yuan, L. | 2022 | 2020 | 249 | 110 | 44.18% | Parental expectation | 3.304 | 0.898 |
| Zheng, S. | 2014 | 2013 | 342 | 138 | 40.35% | Parental expectation | 3.030 | 0.910 |
| Yang, X.Y. | 2008 | 2006 | 375 | 186 | 49.60% | Parental expectation | 2.980 | 0.910 |
| Zhang, Y.Z. | 2016 | 2015 | 348 | 113 | 32.47% | Parental expectation | 2.930 | 0.916 |
| Tang, H.Y. | 2014 | 2012 | 275 | 69 | 25.09% | Parental expectation | 2.960 | 0.925 |
| Wang, M.M. | 2012 | 2010 | 80 | 27 | 33.75% | Parental expectation | 2.950 | 0.928 |
| Chen, H. | 2014 | 2012 | 383 | 205 | 53.52% | Parental expectation | 2.670 | 0.942 |
| Liu, Y.L. | 2016 | 2014 | 771 | 327 | 42.41% | Parental expectation | 1.858 | 0.944 |
| Kang, H.J. | 2012 | 2010 | 228 | 80 | 35.09% | Parental expectation | 3.002 | 0.944 |
| Su, Y.J. | 2010 | 2009 | 426 | 156 | 36.62% | Parental expectation | 2.932 | 0.946 |
| Li, Y.L. | 2015 | 2013 | 285 | 101 | 35.44% | Parental expectation | 2.987 | 0.948 |
| Yu, S.J. | 2008 | 2007 | 419 | 181 | 43.20% | Parental expectation | 2.960 | 0.950 |
| Kuang, L.P. | 2017 | 2015 | 1097 | 373 | 34.00% | Parental expectation | 2.748 | 0.984 |
| Sun, L.L. | 2018 | 2017 | 502 | 147 | 29.28% | Parental expectation | 3.110 | 0.996 |
| Wang, Z.P. | 2021 | 2020 | 505 | 260 | 51.49% | Parental expectation | 2.215 | 0.997 |
| Guo, H.M. | 2012 | 2010 | 761 | N/A | 41.10% | Parental expectation | 3.146 | 1.004 |
| Li, S.N. | 2020 | 2019 | 500 | 79 | 15.80% | Parental expectation | 2.750 | 1.010 |
| Hao, Y.Q. | 2021 | 2020 | 529 | 120 | 22.68% | Parental expectation | 2.672 | 1.013 |
| Wang, Z.H. | 2016 | 2014 | 230 | 74 | 32.17% | Parental expectation | 3.740 | 1.040 |
| He, Q. | 2012 | 2011 | 500 | 377 | 75.40% | Parental expectation | 2.996 | 1.044 |
| Zhang, P. | 2006 | 2005 | 1197 | 572 | 47.79% | Personal standard | 3.061 | 0.400 |
| Jin, Z. | 2012 | 2011 | 174 | 96 | 55.17% | Personal standard | 3.199 | 0.403 |
| Huang, F. | 2007 | 2006 | 362 | 125 | 34.53% | Personal standard | 3.070 | 0.450 |
| Zheng, X.H. | 2011 | 2010 | 755 | 301 | 39.87% | Personal standard | 2.400 | 0.492 |
| Yuan, Y. | 2014 | 2012 | 120 | 17 | 14.17% | Personal standard | 3.070 | 0.520 |
| Yu, Z.X. | 2017 | 2015 | 201 | 54 | 26.87% | Personal standard | 2.901 | 0.550 |
| Wang, W.J. | 2013 | 2011 | 208 | 86 | 41.35% | Personal standard | 3.260 | 0.550 |
| Wang, Y. | 2016 | 2015 | 402 | 0 | 0.00% | Personal standard | 3.004 | 0.556 |
| Chengjiang Han | 2022 | 2021 | 243 | 203 | 83.54% | Personal standard | 3.510 | 0.560 |
| Zhong, Y.H. | 2010 | 2009 | 296 | 206 | 69.59% | Personal standard | 2.970 | 0.570 |
| Wang, J. | 2012 | 2010 | 127 | 0 | 0.00% | Personal standard | 3.490 | 0.580 |
| Bian, Z.W. | 2017 | 2015 | 346 | 154 | 44.51% | Personal standard | 3.223 | 0.585 |
| Zhang, L.G. | 2011 | 2009 | 297 | 144 | 48.48% | Personal standard | 2.350 | 0.600 |
| Zhang, L.G. | 2010 | 2009 | 224 | 97 | 43.30% | Personal standard | 2.274 | 0.634 |
| Li, L. | 2006 | 2005 | 392 | 104 | 26.53% | Personal standard | 2.954 | 0.656 |
| Wang, L.K. | 2010 | 2009 | 792 | 301 | 38.01% | Personal standard | 2.990 | 0.665 |
| Zhang, J.Y. | 2021 | 2020 | 218 | 110 | 50.46% | Personal standard | 3.080 | 0.670 |
| Yang, Y.Y. | 2012 | 2011 | 441 | 175 | 39.68% | Personal standard | 2.970 | 0.673 |
| Zi, F. | 2005 | 2003 | 270 | 270 | 100.00% | Personal standard | 3.234 | 0.678 |
| Ye, Y.S. | 2010 | 2009 | 988 | N/A | 41.10% | Personal standard | 3.180 | 0.680 |
| Sun, J. | 2011 | 2009 | 486 | N/A | 41.10% | Personal standard | 3.180 | 0.680 |
| Shi, B. | 2009 | 2007 | 700 | 237 | 33.86% | Personal standard | 3.070 | 0.700 |
| He, L.T. | 2023 | 2022 | 496 | 116 | 23.39% | Personal standard | 3.078 | 0.700 |
| Yang, H.F. | 2004 | 2002 | 505 | 276 | 54.65% | Personal standard | 3.480 | 0.710 |
| Zhang, J.Y. | 2017 | 2014 | 266 | 86 | 32.33% | Personal standard | 3.283 | 0.717 |
| Guo, K.Y. | 2023 | 2022 | 1006 | 401 | 39.86% | Personal standard | 2.930 | 0.730 |
| Yang, S.H. | 2022 | 2021 | 505 | 217 | 42.97% | Personal standard | 3.247 | 0.732 |
| Wang, C.N. | 2008 | 2007 | 189 | 63 | 33.33% | Personal standard | 2.380 | 0.740 |
| Guo, H.M. | 2012 | 2010 | 761 | N/A | 41.10% | Personal standard | 2.500 | 0.740 |
| Peng, C. | 2014 | 2013 | 475 | 252 | 53.05% | Personal standard | 3.145 | 0.743 |
| Hao, Y.Q. | 2021 | 2020 | 529 | 120 | 22.68% | Personal standard | 3.040 | 0.751 |
| Li, Y.L. | 2015 | 2013 | 285 | 101 | 35.44% | Personal standard | 3.092 | 0.763 |
| Tong, Y.H. | 2006 | 2004 | 257 | 126 | 49.03% | Personal standard | 3.537 | 0.765 |
| Ren, X.M. | 2019 | 2018 | 498 | 193 | 38.76% | Personal standard | 3.100 | 0.767 |
| Wang, M.M. | 2012 | 2010 | 80 | 27 | 33.75% | Personal standard | 2.985 | 0.770 |
| Yang, X.Y. | 2008 | 2006 | 375 | 186 | 49.60% | Personal standard | 3.120 | 0.780 |
| Tang, H.Y. | 2014 | 2012 | 275 | 69 | 25.09% | Personal standard | 2.976 | 0.788 |
| Liu, Y.L. | 2016 | 2014 | 771 | 327 | 42.41% | Personal standard | 3.012 | 0.792 |
| Yuqing Song | 2008 | 2006 | 961 | 525 | 54.63% | Personal standard | 3.556 | 0.796 |
| Li, S.N. | 2020 | 2019 | 500 | 79 | 15.80% | Personal standard | 2.980 | 0.800 |
| Sun, L.L. | 2018 | 2017 | 502 | 147 | 29.28% | Personal standard | 3.250 | 0.802 |
| Zheng, S. | 2014 | 2013 | 342 | 138 | 40.35% | Personal standard | 2.980 | 0.805 |
| Song, Y.Q. | 2017 | 2015 | 189 | 87 | 46.03% | Personal standard | 3.200 | 0.810 |
| Yuan, L. | 2022 | 2020 | 249 | 110 | 44.18% | Personal standard | 3.187 | 0.817 |
| He, Q. | 2012 | 2011 | 500 | 377 | 75.40% | Personal standard | 3.230 | 0.820 |
| Li, R. | 2008 | 2007 | 306 | 152 | 49.67% | Personal standard | 3.667 | 0.827 |
| Zhang, Y.Z. | 2016 | 2015 | 348 | 113 | 32.47% | Personal standard | 3.390 | 0.840 |
| Chen, H. | 2014 | 2012 | 383 | 205 | 53.52% | Personal standard | 2.808 | 0.843 |
| Wang, Z.H. | 2016 | 2014 | 230 | 74 | 32.17% | Personal standard | 2.967 | 0.850 |
| Yu, S.J. | 2008 | 2007 | 419 | 181 | 43.20% | Personal standard | 3.090 | 0.850 |
| Su, Y.J. | 2010 | 2009 | 426 | 156 | 36.62% | Personal standard | 3.041 | 0.857 |
| Kuang, L.P. | 2017 | 2015 | 1097 | 373 | 34.00% | Personal standard | 2.857 | 0.920 |
| Kang, H.J. | 2012 | 2010 | 228 | 80 | 35.09% | Personal standard | 2.990 | 0.942 |
| Wang, Z.P. | 2021 | 2020 | 505 | 260 | 51.49% | Personal standard | 3.777 | 1.080 |
| Jin, Z. | 2012 | 2011 | 174 | 96 | 55.17% | Organization | 3.845 | 0.367 |
| Zhang, P. | 2006 | 2005 | 1197 | 572 | 47.79% | Organization | 3.937 | 0.390 |
| Huang, F. | 2007 | 2006 | 362 | 125 | 34.53% | Organization | 3.130 | 0.480 |
| Yu, Z.X. | 2017 | 2015 | 201 | 54 | 26.87% | Organization | 3.964 | 0.500 |
| Chengjiang Han | 2022 | 2021 | 243 | 203 | 83.54% | Organization | 3.980 | 0.540 |
| Yuan, Y. | 2014 | 2012 | 120 | 17 | 14.17% | Organization | 3.760 | 0.550 |
| Zhong, Y.H. | 2010 | 2009 | 296 | 206 | 69.59% | Organization | 3.800 | 0.590 |
| Wang, W.J. | 2013 | 2011 | 208 | 86 | 41.35% | Organization | 3.940 | 0.590 |
| Ye, Y.S. | 2010 | 2009 | 988 | N/A | 41.10% | Organization | 4.060 | 0.590 |
| Sun, J. | 2011 | 2009 | 486 | N/A | 41.10% | Organization | 4.060 | 0.590 |
| Zhang, J.Y. | 2017 | 2014 | 266 | 86 | 32.33% | Organization | 3.867 | 0.600 |
| Wang, J. | 2012 | 2010 | 127 | 0 | 0.00% | Organization | 3.980 | 0.610 |
| Shi, B. | 2009 | 2007 | 700 | 237 | 33.86% | Organization | 4.020 | 0.610 |
| Zi, F. | 2005 | 2003 | 270 | 270 | 100.00% | Organization | 4.254 | 0.610 |
| Song, Y.Q. | 2017 | 2015 | 189 | 87 | 46.03% | Organization | 3.868 | 0.625 |
| Peng, C. | 2014 | 2013 | 475 | 252 | 53.05% | Organization | 3.223 | 0.638 |
| Su, Y.J. | 2010 | 2009 | 426 | 156 | 36.62% | Organization | 4.385 | 0.640 |
| Zheng, X.H. | 2011 | 2010 | 755 | 301 | 39.87% | Organization | 3.800 | 0.645 |
| Guo, K.Y. | 2023 | 2022 | 1006 | 401 | 39.86% | Organization | 3.730 | 0.650 |
| He, L.T. | 2023 | 2022 | 496 | 116 | 23.39% | Organization | 3.953 | 0.657 |
| Tang, H.Y. | 2014 | 2012 | 275 | 69 | 25.09% | Organization | 4.092 | 0.675 |
| Wang, Y. | 2016 | 2015 | 402 | 0 | 0.00% | Organization | 3.717 | 0.688 |
| Yang, S.H. | 2022 | 2021 | 505 | 217 | 42.97% | Organization | 3.840 | 0.688 |
| Zhang, Y.Z. | 2016 | 2015 | 348 | 113 | 32.47% | Organization | 3.900 | 0.695 |
| Wang, L.K. | 2010 | 2009 | 792 | 301 | 38.01% | Organization | 3.640 | 0.697 |
| Tong, Y.H. | 2006 | 2004 | 257 | 126 | 49.03% | Organization | 3.978 | 0.698 |
| Zhang, J.Y. | 2021 | 2020 | 218 | 110 | 50.46% | Organization | 3.630 | 0.700 |
| Yuqing Song | 2008 | 2006 | 961 | 525 | 54.63% | Organization | 3.625 | 0.702 |
| Li, L. | 2006 | 2005 | 392 | 104 | 26.53% | Organization | 3.763 | 0.712 |
| Bian, Z.W. | 2017 | 2015 | 346 | 154 | 44.51% | Organization | 3.783 | 0.713 |
| Li, S.N. | 2020 | 2019 | 500 | 79 | 15.80% | Organization | 3.958 | 0.717 |
| Yang, X.Y. | 2008 | 2006 | 375 | 186 | 49.60% | Organization | 3.980 | 0.720 |
| Ren, X.M. | 2019 | 2018 | 498 | 193 | 38.76% | Organization | 4.000 | 0.722 |
| Li, Y.L. | 2015 | 2013 | 285 | 101 | 35.44% | Organization | 4.024 | 0.727 |
| Chen, H. | 2014 | 2012 | 383 | 205 | 53.52% | Organization | 3.945 | 0.740 |
| Sun, L.L. | 2018 | 2017 | 502 | 147 | 29.28% | Organization | 3.880 | 0.750 |
| Hao, Y.Q. | 2021 | 2020 | 529 | 120 | 22.68% | Organization | 3.732 | 0.759 |
| Guo, H.M. | 2012 | 2010 | 761 | N/A | 41.10% | Organization | 4.038 | 0.760 |
| Yuan, L. | 2022 | 2020 | 249 | 110 | 44.18% | Organization | 3.882 | 0.762 |
| Kang, H.J. | 2012 | 2010 | 228 | 80 | 35.09% | Organization | 4.097 | 0.768 |
| Zheng, S. | 2014 | 2013 | 342 | 138 | 40.35% | Organization | 3.283 | 0.772 |
| Li, R. | 2008 | 2007 | 306 | 152 | 49.67% | Organization | 4.180 | 0.775 |
| He, Q. | 2012 | 2011 | 500 | 377 | 75.40% | Organization | 4.035 | 0.777 |
| Zhang, L.G. | 2011 | 2009 | 297 | 144 | 48.48% | Organization | 3.050 | 0.790 |
| Kuang, L.P. | 2017 | 2015 | 1097 | 373 | 34.00% | Organization | 3.908 | 0.790 |
| Liu, Y.L. | 2016 | 2014 | 771 | 327 | 42.41% | Organization | 3.842 | 0.800 |
| Yu, S.J. | 2008 | 2007 | 419 | 181 | 43.20% | Organization | 3.980 | 0.800 |
| Yang, Y.Y. | 2012 | 2011 | 441 | 175 | 39.68% | Organization | 3.500 | 0.802 |
| Wang, Z.H. | 2016 | 2014 | 230 | 74 | 32.17% | Organization | 4.017 | 0.833 |
| Zhang, L.G. | 2010 | 2009 | 224 | 97 | 43.30% | Organization | 3.055 | 0.841 |
| Wang, M.M. | 2012 | 2010 | 80 | 27 | 33.75% | Organization | 3.882 | 0.857 |
| Wang, C.N. | 2008 | 2007 | 189 | 63 | 33.33% | Organization | 3.040 | 0.860 |
| Yang, H.F. | 2004 | 2002 | 505 | 276 | 54.65% | Organization | 3.750 | 0.870 |
| Wang, Z.P. | 2021 | 2020 | 505 | 260 | 51.49% | Organization | 3.664 | 1.035 |
| Yang, H.F. | 2004 | 2002 | 505 | 276 | 54.65% | Overall perfectionism | 3.050 | 0.490 |
| Zi, F. | 2005 | 2003 | 270 | 270 | 100.00% | Overall perfectionism | 3.060 | 0.750 |
| Tong, Y.H. | 2006 | 2004 | 257 | 126 | 49.03% | Overall perfectionism | 3.083 | 0.861 |
| Zhang, P. | 2006 | 2005 | 1197 | 572 | 47.79% | Overall perfectionism | 3.047 | 0.419 |
| Li, L. | 2006 | 2005 | 392 | 104 | 26.53% | Overall perfectionism | 2.243 | 0.425 |
| Chang, B.R. | 2008 | 2006 | 701 | 388 | 55.35% | Overall perfectionism | 2.402 | 0.294 |
| Huang, F. | 2007 | 2006 | 362 | 125 | 34.53% | Overall perfectionism | 2.880 | 0.320 |
| Ma, J. | 2008 | 2006 | 1245 | 592 | 47.55% | Overall perfectionism | 2.988 | 0.504 |
| Yang, X.Y. | 2008 | 2006 | 375 | 186 | 49.60% | Overall perfectionism | 3.070 | 0.510 |
| Yuqing Song | 2008 | 2006 | 961 | 525 | 54.63% | Overall perfectionism | 2.829 | 0.688 |
| Li, R. | 2008 | 2007 | 306 | 152 | 49.67% | Overall perfectionism | 3.004 | 0.531 |
| Shi, B. | 2009 | 2007 | 700 | 237 | 33.86% | Overall perfectionism | 2.780 | 0.540 |
| Wang, C.N. | 2008 | 2007 | 189 | 63 | 33.33% | Overall perfectionism | 2.160 | 0.570 |
| Yu, S.J. | 2008 | 2007 | 419 | 181 | 43.20% | Overall perfectionism | 3.062 | 0.912 |
| Ye, Y.S. | 2010 | 2009 | 988 | N/A | 41.10% | Overall perfectionism | 3.110 | 0.420 |
| Sun, J. | 2011 | 2009 | 486 | N/A | 41.10% | Overall perfectionism | 3.110 | 0.420 |
| Zhong, Y.H. | 2010 | 2009 | 296 | 206 | 69.59% | Overall perfectionism | 2.947 | 0.443 |
| Su, Y.J. | 2010 | 2009 | 426 | 156 | 36.62% | Overall perfectionism | 3.039 | 0.520 |
| Wang, L.K. | 2010 | 2009 | 792 | 301 | 38.01% | Overall perfectionism | 2.956 | 0.537 |
| Zhang, L.G. | 2010 | 2009 | 224 | 97 | 43.30% | Overall perfectionism | 2.150 | 0.670 |
| Zhang, L.G. | 2011 | 2009 | 297 | 144 | 48.48% | Overall perfectionism | 2.215 | 0.700 |
| Chen, G.X. | 2011 | 2010 | 955 | 146 | 15.29% | Overall perfectionism | 2.948 | 0.386 |
| Wang, J. | 2012 | 2010 | 127 | 0 | 0.00% | Overall perfectionism | 3.130 | 0.510 |
| Wang, M.M. | 2012 | 2010 | 80 | 27 | 33.75% | Overall perfectionism | 2.953 | 0.516 |
| Guo, H.M. | 2012 | 2010 | 761 | N/A | 41.10% | Overall perfectionism | 2.190 | 0.570 |
| Kang, H.J. | 2012 | 2010 | 228 | 80 | 35.09% | Overall perfectionism | 3.038 | 0.580 |
| Zheng, X.H. | 2011 | 2010 | 755 | 301 | 39.87% | Overall perfectionism | 2.554 | 0.611 |
| Zhao, X.J. | 2012 | 2010 | 267 | 79 | 29.59% | Overall perfectionism | 2.952 | 0.613 |
| Wang, W.J. | 2013 | 2011 | 208 | 86 | 41.35% | Overall perfectionism | 2.420 | 0.350 |
| Jin, Z. | 2012 | 2011 | 174 | 96 | 55.17% | Overall perfectionism | 3.041 | 0.427 |
| Qin, F. | 2013 | 2011 | 269 | 113 | 42.01% | Overall perfectionism | 3.211 | 0.456 |
| Yang, Y.Y. | 2012 | 2011 | 441 | 175 | 39.68% | Overall perfectionism | 2.950 | 0.497 |
| He, Q. | 2012 | 2011 | 500 | 377 | 75.40% | Overall perfectionism | 3.150 | 0.560 |
| Li, Y.C. | 2013 | 2011 | 267 | 131 | 49.06% | Overall perfectionism | 3.347 | 0.699 |
| Tang, H.Y. | 2014 | 2012 | 275 | 69 | 25.09% | Overall perfectionism | 2.990 | 0.481 |
| Yuan, Y. | 2014 | 2012 | 120 | 17 | 14.17% | Overall perfectionism | 2.970 | 0.600 |
| Chen, H. | 2014 | 2012 | 383 | 205 | 53.52% | Overall perfectionism | 2.851 | 0.846 |
| Li, Y.L. | 2015 | 2013 | 285 | 101 | 35.44% | Overall perfectionism | 3.112 | 0.164 |
| Zheng, S. | 2014 | 2013 | 342 | 138 | 40.35% | Overall perfectionism | 3.030 | 0.539 |
| Peng, C. | 2014 | 2013 | 475 | 252 | 53.05% | Overall perfectionism | 3.154 | 0.558 |
| Zhang, J.Y. | 2017 | 2014 | 266 | 86 | 32.33% | Overall perfectionism | 3.181 | 0.437 |
| Wang, Z.H. | 2016 | 2014 | 230 | 74 | 32.17% | Overall perfectionism | 3.007 | 0.496 |
| Liu, Y.L. | 2016 | 2014 | 771 | 327 | 42.41% | Overall perfectionism | 3.040 | 0.532 |
| Yu, Z.X. | 2017 | 2015 | 201 | 54 | 26.87% | Overall perfectionism | 3.381 | 0.350 |
| Wang, Y. | 2016 | 2015 | 402 | 0 | 0.00% | Overall perfectionism | 2.962 | 0.408 |
| Bian, Z.W. | 2017 | 2015 | 346 | 154 | 44.51% | Overall perfectionism | 3.169 | 0.446 |
| Zhang, Y.Z. | 2016 | 2015 | 348 | 113 | 32.47% | Overall perfectionism | 3.230 | 0.593 |
| Song, Y.Q. | 2017 | 2015 | 189 | 87 | 46.03% | Overall perfectionism | 3.226 | 0.824 |
| Kuang, L.P. | 2017 | 2015 | 1097 | 373 | 34.00% | Overall perfectionism | 2.934 | 0.896 |
| Ge, C. | 2017 | 2016 | 364 | 180 | 49.45% | Overall perfectionism | 2.944 | 0.465 |
| Xie, H.M. | 2018 | 2016 | 1589 | 595 | 37.44% | Overall perfectionism | 3.958 | 0.581 |
| Miao, L.T. | 2018 | 2016 | 578 | 316 | 54.67% | Overall perfectionism | 2.879 | 0.732 |
| Sun, L.L. | 2018 | 2017 | 502 | 147 | 29.28% | Overall perfectionism | 3.114 | 0.868 |
| Miao, H.X. | 2020 | 2018 | 172 | 36 | 20.93% | Overall perfectionism | 2.752 | 0.379 |
| Ren, X.M. | 2019 | 2018 | 498 | 193 | 38.76% | Overall perfectionism | 3.150 | 0.581 |
| Cao, H. | 2020 | 2018 | 271 | 101 | 37.27% | Overall perfectionism | 2.961 | 0.604 |
| Wang, Z.Y. | 2020 | 2018 | 568 | 242 | 42.61% | Overall perfectionism | 3.097 | 0.745 |
| Tang, H.Y. | 2021 | 2019 | 409 | 137 | 33.50% | Overall perfectionism | 3.164 | 0.580 |
| Linlin Feng | 2021 | 2019 | 525 | 198 | 37.71% | Overall perfectionism | 3.323 | 0.717 |
| Li, S.N. | 2020 | 2019 | 500 | 79 | 15.80% | Overall perfectionism | 3.010 | 0.850 |
| Liu, Y. | 2022 | 2020 | 522 | 191 | 36.59% | Overall perfectionism | 3.293 | 0.544 |
| Yuan, L. | 2022 | 2020 | 249 | 110 | 44.18% | Overall perfectionism | 3.267 | 0.624 |
| Hao, Y.Q. | 2021 | 2020 | 529 | 120 | 22.68% | Overall perfectionism | 2.984 | 0.661 |
| Zhang, J.Y. | 2021 | 2020 | 218 | 110 | 50.46% | Overall perfectionism | 3.029 | 0.758 |
| Luming Liu | 2022 | 2020 | 1865 | 793 | 42.52% | Overall perfectionism | 2.259 | 0.784 |
| Wang, Z.P. | 2021 | 2020 | 505 | 260 | 51.49% | Overall perfectionism | 2.931 | 0.961 |
| Chen, H.H. | 2022 | 2021 | 1264 | 419 | 33.15% | Overall perfectionism | 2.186 | 0.423 |
| Luo, J. | 2022 | 2021 | 589 | 236 | 40.07% | Overall perfectionism | 3.050 | 0.423 |
| Yang, Z. | 2023 | 2021 | 844 | 247 | 29.27% | Overall perfectionism | 3.326 | 0.549 |
| Yang, S.H. | 2022 | 2021 | 505 | 217 | 42.97% | Overall perfectionism | 3.260 | 0.661 |
| Chengjiang Han | 2022 | 2021 | 243 | 203 | 83.54% | Overall perfectionism | 3.336 | 0.732 |
| He, L.T. | 2023 | 2022 | 496 | 116 | 23.39% | Overall perfectionism | 3.086 | 0.453 |
| Yang, X.Y. | 2022 | 2022 | 318 | 144 | 45.28% | Overall perfectionism | 3.078 | 0.576 |
| Guo, K.Y. | 2023 | 2022 | 1006 | 401 | 39.86% | Overall perfectionism | 3.127 | 0.626 |
| Jin, Z. | 2012 | 2011 | 174 | 96 | 55.17% | Doubts about actions | 3.065 | 0.306 |
| Zhang, P. | 2006 | 2005 | 1197 | 572 | 47.79% | Doubts about actions | 3.138 | 0.440 |
| Huang, F. | 2007 | 2006 | 362 | 125 | 34.53% | Doubts about actions | 2.790 | 0.520 |
| Zheng, X.H. | 2011 | 2010 | 755 | 301 | 39.87% | Doubts about actions | 2.190 | 0.578 |
| Yu, Z.X. | 2017 | 2015 | 201 | 54 | 26.87% | Doubts about actions | 3.240 | 0.580 |
| Yuan, Y. | 2014 | 2012 | 120 | 17 | 14.17% | Doubts about actions | 3.260 | 0.640 |
| Bian, Z.W. | 2017 | 2015 | 346 | 154 | 44.51% | Doubts about actions | 3.230 | 0.665 |
| Zhang, L.G. | 2010 | 2009 | 224 | 97 | 43.30% | Doubts about actions | 2.142 | 0.669 |
| Zhang, L.G. | 2011 | 2009 | 297 | 144 | 48.48% | Doubts about actions | 2.270 | 0.670 |
| Wang, W.J. | 2013 | 2011 | 208 | 86 | 41.35% | Doubts about actions | 3.100 | 0.670 |
| Wang, J. | 2012 | 2010 | 127 | 0 | 0.00% | Doubts about actions | 3.320 | 0.690 |
| Yang, Y.Y. | 2012 | 2011 | 441 | 175 | 39.68% | Doubts about actions | 2.940 | 0.693 |
| Wang, Y. | 2016 | 2015 | 402 | 0 | 0.00% | Doubts about actions | 3.070 | 0.695 |
| Wang, L.K. | 2010 | 2009 | 792 | 301 | 38.01% | Doubts about actions | 2.960 | 0.705 |
| Yuqing Song | 2008 | 2006 | 961 | 525 | 54.63% | Doubts about actions | 2.993 | 0.718 |
| Yang, S.H. | 2022 | 2021 | 505 | 217 | 42.97% | Doubts about actions | 3.355 | 0.723 |
| Zhang, J.Y. | 2017 | 2014 | 266 | 86 | 32.33% | Doubts about actions | 3.225 | 0.725 |
| Zhong, Y.H. | 2010 | 2009 | 296 | 206 | 69.59% | Doubts about actions | 2.930 | 0.740 |
| Guo, K.Y. | 2023 | 2022 | 1006 | 401 | 39.86% | Doubts about actions | 3.340 | 0.750 |
| Ye, Y.S. | 2010 | 2009 | 988 | N/A | 41.10% | Doubts about actions | 3.170 | 0.760 |
| Sun, J. | 2011 | 2009 | 486 | N/A | 41.10% | Doubts about actions | 3.170 | 0.760 |
| Wang, Z.H. | 2016 | 2014 | 230 | 74 | 32.17% | Doubts about actions | 3.175 | 0.775 |
| Wang, C.N. | 2008 | 2007 | 189 | 63 | 33.33% | Doubts about actions | 2.010 | 0.780 |
| Yang, X.Y. | 2008 | 2006 | 375 | 186 | 49.60% | Doubts about actions | 3.020 | 0.790 |
| Li, L. | 2006 | 2005 | 392 | 104 | 26.53% | Doubts about actions | 2.830 | 0.795 |
| Shi, B. | 2009 | 2007 | 700 | 237 | 33.86% | Doubts about actions | 2.730 | 0.800 |
| Yang, H.F. | 2004 | 2002 | 505 | 276 | 54.65% | Doubts about actions | 2.890 | 0.800 |
| Zhang, J.Y. | 2021 | 2020 | 218 | 110 | 50.46% | Doubts about actions | 3.050 | 0.800 |
| Chengjiang Han | 2022 | 2021 | 243 | 203 | 83.54% | Doubts about actions | 3.120 | 0.800 |
| Zhang, Y.Z. | 2016 | 2015 | 348 | 113 | 32.47% | Doubts about actions | 3.390 | 0.810 |
| Liu, Y.L. | 2016 | 2014 | 771 | 327 | 42.41% | Doubts about actions | 3.140 | 0.830 |
| Peng, C. | 2014 | 2013 | 475 | 252 | 53.05% | Doubts about actions | 3.115 | 0.835 |
| Sun, L.L. | 2018 | 2017 | 502 | 147 | 29.28% | Doubts about actions | 3.380 | 0.840 |
| Tang, H.Y. | 2014 | 2012 | 275 | 69 | 25.09% | Doubts about actions | 2.982 | 0.842 |
| Zheng, S. | 2014 | 2013 | 342 | 138 | 40.35% | Doubts about actions | 3.120 | 0.850 |
| Ren, X.M. | 2019 | 2018 | 498 | 193 | 38.76% | Doubts about actions | 3.180 | 0.853 |
| He, Q. | 2012 | 2011 | 500 | 377 | 75.40% | Doubts about actions | 3.240 | 0.860 |
| Li, S.N. | 2020 | 2019 | 500 | 79 | 15.80% | Doubts about actions | 3.270 | 0.860 |
| Kang, H.J. | 2012 | 2010 | 228 | 80 | 35.09% | Doubts about actions | 3.060 | 0.865 |
| Li, Y.L. | 2015 | 2013 | 285 | 101 | 35.44% | Doubts about actions | 3.189 | 0.873 |
| Hao, Y.Q. | 2021 | 2020 | 529 | 120 | 22.68% | Doubts about actions | 3.188 | 0.879 |
| Wang, M.M. | 2012 | 2010 | 80 | 27 | 33.75% | Doubts about actions | 3.008 | 0.893 |
| Zi, F. | 2005 | 2003 | 270 | 270 | 100.00% | Doubts about actions | 3.221 | 0.897 |
| Kuang, L.P. | 2017 | 2015 | 1097 | 373 | 34.00% | Doubts about actions | 3.200 | 0.900 |
| Yuan, L. | 2022 | 2020 | 249 | 110 | 44.18% | Doubts about actions | 3.445 | 0.933 |
| Guo, H.M. | 2012 | 2010 | 761 | N/A | 41.10% | Doubts about actions | 3.033 | 0.950 |
| Chen, H. | 2014 | 2012 | 383 | 205 | 53.52% | Doubts about actions | 2.855 | 0.955 |
| Su, Y.J. | 2010 | 2009 | 426 | 156 | 36.62% | Doubts about actions | 3.092 | 0.961 |
| Li, R. | 2008 | 2007 | 306 | 152 | 49.67% | Doubts about actions | 3.010 | 0.974 |
| Wang, Z.P. | 2021 | 2020 | 505 | 260 | 51.49% | Doubts about actions | 2.250 | 1.009 |
| Yu, S.J. | 2008 | 2007 | 419 | 181 | 43.20% | Doubts about actions | 3.050 | 1.050 |
| He, L.T. | 2023 | 2022 | 496 | 116 | 23.39% | Doubts about actions | 4.618 | 1.050 |
| Song, Y.Q. | 2017 | 2015 | 189 | 87 | 46.03% | Doubts about actions | 3.195 | 1.195 |
| Tong, Y.H. | 2006 | 2004 | 257 | 126 | 49.03% | Doubts about actions | 2.823 | 1.343 |

*Note. Y* indicates the year of publication. *Year* refers to the year of data collection. *N* represents the total sample size, and *n* refers to the number of male participants.

**Appendix Two: Meta-analysis References**

Bian ZW (2017) A study on the correlation between procrastination and perfectionism in college students. J Tonghua Norm Univ 38(2):141–145. <https://doi.org/10.13877/j.cnki.cn22-1284.2017.02.036>

Cao H, Li D, Fang F (2020) Perfectionism and trait depression in college students: The mediating role of psychological resilience. Think Tank Era 2020(14):120–123.

Chang BR, Fang JD (2008) The study on relationship of perfectionism, self-esteem, self-consistency and congruence and suicidal intention in undergraduates. Chin J Health Psychol 16(6):634–637. <https://doi.org/10.13342/j.cnki.cjhp.2008.06.022>

Chen GX (2011) The relationship among perfectionism, rumination and depression of college students. Master’s thesis, Guangxi Normal Univ.

Chen H, Zhang HY (2014) Research on the relationship between perfectionism and depression in college students: The moderating role of self-concept. J Open Univ Shaanxi 16(2):59–64.

Chen HH, Yang YL, Li Y, Yan WS (2022) Perfectionism and affect balance among college students: The chain mediating roles of psychological resilience and rumination. Chin J Health Psychol 30(2):247–252. <https://doi.org/10.13342/j.cnki.cjhp.2022.02.018>

Feng L, Zhang L, Zhong H (2021) Perceived parenting styles and mental health: The multiple mediation effect of perfectionism and altruistic behavior. Psychol Res Behav Manag 14:1157–1170. <https://doi.org/10.2147/PRBM.S318446>

Ge C, Wang H (2017) The relationship between perfectionism and learning motivation in college students: The mediating role of self-esteem. J Xinyang Norm Univ (Philos Soc Sci) 37(2):63–68. <https://doi.org/10.3969/j.issn.1003-0964.2017.02.008>

Guo HM, Yu F, Huang H, Qian C (2012) Mediation effect of self-efficacy on relationship between perfectionism and psychological well-being in medical students. Chin J Public Health 28(6):840–842.

Guo KY (2022) The relationship between perfectionism and social anxiety in college students: The mediating role of social self-efficacy and reinforcement sensitivity. Master’s thesis, Sichuan Normal Univ.

Hao YQ (2021) The relationship and intervention of perfectionism, emotional resilience and sleep quality among college students. Master’s thesis, Hebei Normal Univ.

Han C, Li F, Lian B, Vencúrik T, Liang W (2022) Relationships between perfectionism, extra training, and academic performance in Chinese collegiate athletes: Mediating role of achievement motivation. Int J Environ Res Public Health 19(17):10764. <https://doi.org/10.3390/ijerph191710764>

He Q (2012) Research on the relation between the perfectionism and subjective well-being in engineering college. Master’s thesis, Tianjin Univ.

He LT, Li HX, Ji XG, Xu ZZ (2023) The mediating role of positive coping style in perfectionism and academic procrastination among undergraduate nursing students. Chin Gen Pract Nurs 21(6):746–750. <https://doi.org/10.12104/j.issn.1674-4748.2023.06.006>

Huang F (2007) Perfectionism tendency and its relationship with mental health in college students. Master’s thesis, Zhengzhou Univ.

Jin Z (2012) Relationship between scores and psychology of perfectionism of P.E. college students. J Phys Educ 19(4):89–91. <https://doi.org/10.16419/j.cnki.42-1684/g8.2012.04.032>

Kang HJ, Chen X (2012) Relationships among college students' perfectionism, interpersonal sensitivity, depression and suicidal intention. Chin J Health Psychol 20(1):110–113. <https://doi.org/10.13342/j.cnki.cjhp.2012.01.032>

Kuang LP, He XM, Zu SM, Feng XG (2017) The influence of negative parenting styles on self-injury behavior: The mediating role of perfectionism. Chin J Spec Educ 2017(11):86–87.

Li L (2006) The influence of perfectionism on self-esteem, interpersonal sensitivity and depression in college students. Master’s thesis, Shanghai Normal Univ.

Li R (2008) Perfectionism personality and its relationship with self-esteem and distress in college students. Master’s thesis, Shaanxi Normal Univ.

Li SN (2020) The relationship between rumination, perfectionism and anxiety in college students and intervention. Master’s thesis, Liaoning Normal Univ.

Li YL, Wu R (2015) The relation between perfectionism and social adaptation of college students: Self-esteem as a mediator. J Jimei Univ (Educ Sci) 16(4):21–28.

Li YC, Wang SY (2013) Research on the relationship between learning motivation and academic procrastination: The moderating role of perfectionism. Youth Res 31(8):85–88.

Liu Y, Liu ZH, Duan HZ, Chen GX, Liu WJ (2022) The influence of insecure attachment on academic procrastination: The mediating role of perfectionism and rumination. Psychol Tech Appl 10(7):407–414. <https://doi.org/10.16842/j.cnki.issn2095-5588.2022.07.003>

Liu YL, Lu GZ, Zhang SC, Jin TL, Zhang YL (2016) Mediating effect of self-esteem on relationship between embarrassment and perfectionism in college students. Chin Ment Health J 30(5):389–394. <https://doi.org/10.3969/j.issn.1000-6729.2016.05.013>

Liu L, Han Y, Lu Z, Cao C, Wang W (2023) The relationship between perfectionism and depressive symptoms among Chinese college students: The mediating roles of self-compassion and impostor syndrome. Curr Psychol. <https://doi.org/10.1007/s12144-022-03036-8>

Luo J, An LN (2022) The influence of perfectionism on academic procrastination in college students: The mediating role of academic self-efficacy. J Guizhou Norm Coll 38(11):77–87. <https://doi.org/10.13391/j.cnki.issn.1674-7798.2022.11.004>

Ma J, Feng ZY, Yang XL (2008) Chinese Frost multidimensional perfectionism scale: A survey of perfectionism under different background in undergraduates. J Army Med Univ 30(23):2223–2225. <https://doi.org/10.16016/j.1000-5404.2008.23.032>

Miao HX, Song GW, Jiang ZW (2020) Perfectionism and professional commitment in college students: The mediating effect of academic self-efficacy. J Adult Educ Coll Hebei Univ 22(4):84–92.

Miao LT et al. (2018) Effect of perfectionism on college students' academic procrastination: The moderated mediation model. Chin J Appl Psychol 24(3):252–260.

Peng C (2014) Research on the relationship between perfectionism, achievement motivation and employment expectation in college students. Master’s thesis, Hunan Normal Univ.

Qin F (2013) Research on perfectionism and its relationship with self-esteem and emotion in college students. China Electr Power Educ 2013(23):119–120. <https://doi.org/10.3969/j.issn.1007-0079.2013.023.060>

Ren XM (2019) The relationship between perfectionism and procrastination in college students: The multiple mediating effects of achievement motivation and coping style. Master’s thesis, Qinghai Normal Univ.

Shi B, Yue CZ, Tang W (2009) The relationship between perfectionism and procrastination. Health Med Res Pract 6(3):28–30.

Song YQ, Luo ZR (2017) The relationship between perfectionism and emotional intelligence, test anxiety of college students. Chin J Health Psychol 25(12):1898–1901. <https://doi.org/10.13342/j.cnki.cjhp.2017.12.038>

Song Y et al. (2008) Depression in college: Depressive symptoms and personality factors in Beijing and Hong Kong college freshmen. Compr Psychiatry 49(5):496–502. <https://doi.org/10.1016/j.comppsych.2008.02.005>

Su YJ (2010) The relationship among perfectionism, achievement goal orientation, future time perspective and academic procrastination in college students. Master’s thesis, Shandong Normal Univ.

Sun J, Ye YS (2011) Investigation on perfectionism and learning adaptability of nursing specialty students. Chin Nurs Res 25(1A):29–30. <https://doi.org/10.3969/j.issn.1009-6493.2011.01.011>

Sun LL (2018) The relationship between perfectionism, psychological resilience and sleep quality in college students. Master’s thesis, Southern Medical Univ.

Tang HY, Linghu SF, Ni L, Huang R, Rao C, Wu JH (2021) The relationship among perfectionism, loneliness and school adaptation of university students: A case study of Guizhou Education University. J Guizhou Educ Univ 37(9):55–61. <https://doi.org/10.13391/j.cnki.issn.1674-7798.2021.09.010>

Tang HY, Qiu X, Chen A, Wang ZJ (2014) The influence of college students' perfectionism on loneliness: The mediating effect of self-esteem. Chin J Health Psychol 22(10):1577–1579. <https://doi.org/10.13342/j.cnki.cjhp.2014.10.056>

Tong YH (2006) A study on perfectionism in college students. J Univ Jinan (Sci Technol) 20(3):277–279.

Wang CN (2008) Research on perfectionism, achievement motivation and their relationship with mental health. Master’s thesis, Liaoning Normal Univ.

Wang J, Wang ZL, Wu L, Zhao ZZ (2012) Investigation on perfectionism and self-monitoring of junior college nursing students in clinical practice. Nurs Pract Res 9(14):601–604. <https://doi.org/10.3969/j.issn.1672-9676.2012.14.059>

Wang LK (2014) Research on the relationship among fear of failure, perfectionism and procrastination in college students. Master’s thesis, Hebei Normal Univ.

Wang MM, Li XY (2012) Research on the correlation between perfectionism and attention bias in college students. J Chifeng Univ (Nat Sci) 28(4):166–169. <https://doi.org/10.13398/j.cnki.issn1673-260x.2012.08.068>

Wang WJ, Sun HW, Wang XF (2013) Discuss the relationship between procrastination behavior and perfectionism. Chin Med Pharm 3(15):69–72.

Wang Y (2017) Testing the mediating effect of perfectionism between family parenting style and eating disorders in female college students. Master’s thesis, Henan Univ.

Wang ZY, Zhao WY, Ma JC (2020) Relationship between perfectionism and academic procrastination in college students: The mediating effect of rumination. Chin J Health Psychol 28(10):1597–1601.

Wang ZH, Yang ZN (2016) Correlation between perfectionism and personality with five kinds of constitutions in college students. J Anhui Univ Chin Med 24(4):57–61. <https://doi.org/10.3969/j.issn.2095-7246.2016.04.006>

Wang ZP (2021) The influence of perfectionism on learning flow experience in college students: The chain mediating effect of self-efficacy and learning motivation. Master’s thesis, Jilin Univ.

Xie HM, Wang JJ, Xu X (2018) Analysis of intermediate relationship among perfectionism, active procrastination and anxiety of normal college students. Chin J Sch Health 39(2):219–222. <https://doi.org/10.16835/j.cnki.1000-9817.2018.02.018>

Yang HF, Zhang XY (2004) Perfectionism and mental health of college students. Chin Ment Health J 18(9):647–648.

Yang SH (2021) The influence of perfectionism on academic procrastination in college students: A moderated mediation effect. Master’s thesis, Wuhan Sports Univ.

Yang XY, Liu JT, Shao SH (2022) The relationship between perfectionism personality and aggressive behavior of college students: Mediation of negative physical self. Psychol Monthly 17(21):16–18. <https://doi.org/10.19738/j.cnki.psy.2022.21.005>

Yang XY, Wu Z, Qi B (2008) The relationship between perfectionism and achievement motivation in 375 college students. Chin Ment Health J 22(10):755–756.

Yang YY (2010) Research on learning procrastination and its relationship with achievement goal orientation and perfectionism in college students. Master’s thesis, Nanjing Normal Univ.

Yang Z (2023) The influence of perfectionism on learning addiction in college students: A multiple mediation model. Master’s thesis, Guangzhou Univ.

Ye YS (2010) Research on the relationship among perfectionism, self-confidence and learning adaptability in nursing students. Master’s thesis, Yanbian Univ.

Yu SJ (2008) Research on the correlation among perfectionism, career decision-making self-efficacy and career indecision in college students. Master’s thesis, Beijing Normal Univ.

Yu ZX, Li YL, Hu Y (2017) The relationship between perfectionism and perceived stress in college students. J Dezhou Univ 33(1):34–41.

Yuan Y, Niu Z, Peng JG, Wu SH, He Y, Feng K (2012) The relationship between implicit/explicit self-esteem and perfectionism in college students. Sci Humanit Soc 20(1):110–113.

Yuan L, Zhang J (2022) A study on the correlation between perfectionism and test anxiety of university students. J Weifang Univ 22(6):104–107.

Zhang JY (2021) The relationship between perfectionism and self-deception in college students: The mediating role of self-esteem and moderating role of self-control. Master’s thesis, Ludong Univ.

Zhang JY, Wang LG, Gao WB (2017) Mediating effect of lifestyle actual-ideal self-discrepancy on the relationship between perfectionism and ego depletion. Chin Gen Pract 20(2):206–209. <https://doi.org/10.3969/j.issn.1007-9572.2017.02.017>

Zhang P (2006) Research on the relationship among perfectionism, learning motivation and mental health in Hunan college students. Master’s thesis, Hunan Normal Univ.

Zhang YZ (2017) Research on the relationship among academic procrastination behavior, perfectionism and self-consciousness in college students. Master’s thesis, Soochow Univ.

Zhang LG, Li L (2011) The relationship between perfectionism and family function among college students. Mod Prev Med 38(22):4654–4656.

Zhang LG (2010) Research on the relationship between state-trait anxiety and perfectionism in local college students. J Shaoxing Univ 30(10):89–92.

Zhao XJ, Li CQ (2012) Research on the correlation among self-esteem, sense of security and perfectionism in college students. Educ Teach Forum 32(4):176–179.

Zheng S (2014) The relationship and intervention among perfectionism, self-esteem and academic procrastination in college students. Master’s thesis, Jiangxi Normal Univ.

Zheng XH (2011) Research on the relationship among procrastination behavior, perfectionism and perceived stress in college students. Master’s thesis, Hebei Univ.

Zhong YH, Tang RX (2010) Research on the relationship between perfectionism and mental health in freshmen. J Jiangxi Univ Sci Technol 31(4):26–31. <https://doi.org/10.13265/j.cnki.jxlgdxxb.2010.04.009>

Zi F, Zhou X (2005) Relative study of perfectionism, shyness and suicidal intention among male sophomores. Chin J Health Psychol 13(4):244–245.

**Appendix Three: Outlier Diagnostics for Overall Perfectionism**

| **Obs** | **rstudent** | **dffits** | **cook.d** | **cov.r** | **tau2.del** | **QE.del** | **hat** | **weight** |
| --- | --- | --- | --- | --- | --- | --- | --- | --- |
| 1 | 0.8625 | 0.2317 | 0.0539 | 1.0799 | 0.0982 | 19592.858 | 0.0673 | 1.3584 |
| 2 | 0.827 | 0.2029 | 0.0413 | 1.0695 | 0.0983 | 19665.943 | 0.0567 | 1.3366 |
| 3 | 0.8387 | 0.188 | 0.0355 | 1.0588 | 0.0982 | 19669.6588 | 0.0478 | 1.3259 |
| 4 | 0.7323 | 0.1665 | 0.0279 | 1.0654 | 0.0985 | 19420.8848 | 0.0492 | 1.363 |
| 5 | -1.9324 | -0.4388 | 0.1854 | 0.9758 | 0.0942 | 18808.0245 | 0.049 | 1.3586 |
| 6 | -1.493 | -0.2827 | 0.0785 | 1.0011 | 0.0961 | 17551.4287 | 0.0346 | 1.3633 |
| 7 | 0.1296 | 0.0269 | 0.0007 | 1.0723 | 0.0992 | 19686.1967 | 0.0413 | 1.3611 |
| 8 | 0.4228 | 0.08 | 0.0065 | 1.0599 | 0.099 | 19628.1971 | 0.0346 | 1.3622 |
| 9 | 0.6875 | 0.1298 | 0.017 | 1.051 | 0.0985 | 19634.8909 | 0.0344 | 1.3554 |
| 10 | -0.0908 | -0.0171 | 0.0003 | 1.0649 | 0.0992 | 19680.2095 | 0.0345 | 1.3582 |
| 11 | 0.4729 | 0.0892 | 0.008 | 1.0582 | 0.0989 | 19670.2266 | 0.0343 | 1.3523 |
| 12 | -0.3036 | -0.0521 | 0.0028 | 1.056 | 0.0991 | 19655.0399 | 0.0287 | 1.3592 |
| 13 | -2.3141 | -0.4342 | 0.178 | 0.9217 | 0.0922 | 19379.5001 | 0.034 | 1.3414 |
| 14 | 0.6571 | 0.1232 | 0.0153 | 1.0515 | 0.0986 | 19670.1029 | 0.0339 | 1.3379 |
| 15 | 0.7037 | 0.1099 | 0.0122 | 1.039 | 0.0985 | 19428.5544 | 0.0238 | 1.3625 |
| 16 | 0.646 | 0.0919 | 0.0085 | 1.037 | 0.0986 | 19574.6972 | 0.0199 | 1.36 |
| 17 | 0.1775 | 0.0277 | 0.0008 | 1.0524 | 0.0992 | 19683.2154 | 0.0237 | 1.3558 |
| 18 | 0.4729 | 0.0737 | 0.0055 | 1.0468 | 0.0989 | 19656.202 | 0.0237 | 1.3562 |
| 19 | 0.2067 | 0.0323 | 0.0011 | 1.0522 | 0.0992 | 19678.0441 | 0.0238 | 1.36 |
| 20 | -2.4545 | -0.3796 | 0.1349 | 0.8964 | 0.0915 | 19402.8137 | 0.0234 | 1.3376 |
| 21 | -2.2847 | -0.3228 | 0.0986 | 0.9113 | 0.0924 | 19390.9923 | 0.0196 | 1.3424 |
| 22 | 0.1257 | 0.0179 | 0.0003 | 1.0489 | 0.0992 | 19677.1255 | 0.0199 | 1.3628 |
| 23 | 0.6485 | 0.0842 | 0.0072 | 1.0332 | 0.0986 | 19664.8352 | 0.0166 | 1.337 |
| 24 | 0.0851 | 0.011 | 0.0001 | 1.0445 | 0.0992 | 19686.0251 | 0.0164 | 1.3201 |
| 25 | -2.4496 | -0.3209 | 0.0962 | 0.889 | 0.0914 | 18413.2057 | 0.0169 | 1.3591 |
| 26 | 0.3563 | 0.0464 | 0.0022 | 1.0419 | 0.099 | 19676.9393 | 0.0167 | 1.3447 |
| 27 | -1.1454 | -0.1629 | 0.0264 | 1.0114 | 0.0974 | 19423.6252 | 0.0198 | 1.3582 |
| 28 | 0.0827 | 0.0108 | 0.0001 | 1.0454 | 0.0992 | 19685.6348 | 0.0167 | 1.3457 |
| 29 | -1.7024 | -0.2088 | 0.0425 | 0.9636 | 0.0953 | 19239.5389 | 0.0148 | 1.3569 |
| 30 | 0.3667 | 0.0479 | 0.0023 | 1.0418 | 0.099 | 19672.3874 | 0.0168 | 1.3506 |
| 31 | 0.8577 | 0.1051 | 0.0111 | 1.0225 | 0.0982 | 19583.151 | 0.0148 | 1.3543 |
| 32 | 0.0767 | 0.01 | 0.0001 | 1.0458 | 0.0992 | 19684.7695 | 0.0168 | 1.3573 |
| 33 | 0.7176 | 0.0939 | 0.0089 | 1.031 | 0.0985 | 19601.3291 | 0.0168 | 1.3563 |
| 34 | 1.2946 | 0.1578 | 0.0247 | 0.9963 | 0.0969 | 19590.8901 | 0.0146 | 1.34 |
| 35 | 0.0943 | 0.0111 | 0.0001 | 1.0424 | 0.0992 | 19683.2524 | 0.0137 | 1.3534 |
| 36 | 0.0303 | 0.0035 | 0 | 1.0417 | 0.0992 | 19686.0262 | 0.0134 | 1.3244 |
| 37 | -0.3467 | -0.0407 | 0.0017 | 1.0387 | 0.099 | 19682.1627 | 0.0136 | 1.3394 |
| 38 | 0.43 | 0.0506 | 0.0026 | 1.0374 | 0.099 | 19380.2467 | 0.0137 | 1.3637 |
| 39 | 0.2217 | 0.0261 | 0.0007 | 1.0412 | 0.0991 | 19676.5621 | 0.0137 | 1.3533 |
| 40 | 0.6186 | 0.073 | 0.0054 | 1.0315 | 0.0987 | 19615.2211 | 0.0137 | 1.3559 |
| 41 | 0.5402 | 0.0693 | 0.0049 | 1.0368 | 0.0988 | 19621.913 | 0.0162 | 1.3551 |
| 42 | 0.0389 | 0.0046 | 0 | 1.0432 | 0.0992 | 19684.0739 | 0.0144 | 1.3503 |
| 43 | 0.1444 | 0.0174 | 0.0003 | 1.043 | 0.0992 | 19667.5907 | 0.0145 | 1.3599 |
| 44 | 1.1893 | 0.1529 | 0.0232 | 1.0049 | 0.0973 | 19401.3109 | 0.0163 | 1.3566 |
| 45 | -0.1046 | -0.0128 | 0.0002 | 1.0432 | 0.0992 | 19686.3017 | 0.0145 | 1.3593 |
| 46 | 0.502 | 0.0645 | 0.0042 | 1.038 | 0.0989 | 19614.4403 | 0.0163 | 1.357 |
| 47 | 0.7514 | 0.0908 | 0.0083 | 1.0269 | 0.0984 | 19612.5129 | 0.0144 | 1.3511 |
| 48 | 0.675 | 0.0854 | 0.0074 | 1.0311 | 0.0985 | 19667.662 | 0.0158 | 1.3167 |
| 49 | -0.2488 | -0.032 | 0.001 | 1.0435 | 0.0991 | 19684.8121 | 0.0162 | 1.3549 |
| 50 | -0.217 | -0.028 | 0.0008 | 1.044 | 0.0991 | 19685.4177 | 0.0163 | 1.3568 |
| 51 | 3.1821 | 0.4449 | 0.1754 | 0.8019 | 0.0866 | 14917.7733 | 0.0191 | 1.3621 |
| 52 | -0.48 | -0.0667 | 0.0045 | 1.0414 | 0.0989 | 19675.9469 | 0.0189 | 1.3522 |
| 53 | 0.27 | 0.0373 | 0.0014 | 1.0457 | 0.0991 | 19673.6783 | 0.0188 | 1.3444 |
| 54 | -1.0059 | -0.1684 | 0.0284 | 1.0277 | 0.0978 | 19614.1643 | 0.0273 | 1.3535 |
| 55 | 0.3319 | 0.0505 | 0.0026 | 1.0489 | 0.0991 | 19645.9013 | 0.0227 | 1.3556 |
| 56 | -0.3283 | -0.0549 | 0.003 | 1.0537 | 0.0991 | 19685.4584 | 0.0271 | 1.3465 |
| 57 | 0.107 | 0.0178 | 0.0003 | 1.0567 | 0.0992 | 19675.5964 | 0.0272 | 1.3515 |
| 58 | 0.2676 | 0.0492 | 0.0025 | 1.0611 | 0.0991 | 19654.7905 | 0.0328 | 1.3536 |
| 59 | 0.7814 | 0.1439 | 0.0208 | 1.0451 | 0.0984 | 19580.933 | 0.0328 | 1.3515 |
| 60 | -0.1713 | -0.0287 | 0.0008 | 1.0559 | 0.0992 | 19686.1919 | 0.0271 | 1.3452 |
| 61 | 0.6317 | 0.128 | 0.0165 | 1.0587 | 0.0986 | 19543.723 | 0.0395 | 1.3572 |
| 62 | 0.5443 | 0.1097 | 0.0122 | 1.0611 | 0.0988 | 19644.8512 | 0.0391 | 1.3435 |
| 63 | -0.3114 | -0.0575 | 0.0033 | 1.0604 | 0.0991 | 19685.8146 | 0.0328 | 1.3536 |
| 64 | -0.165 | -0.0302 | 0.0009 | 1.0613 | 0.0991 | 19686.1165 | 0.0323 | 1.3292 |
| 65 | -2.8677 | -0.5819 | 0.3074 | 0.8595 | 0.0888 | 17838.0021 | 0.0396 | 1.3604* |
| 66 | -0.4799 | -0.088 | 0.0078 | 1.0558 | 0.0989 | 19683.3466 | 0.0325 | 1.34 |
| 67 | -3.1554 | -0.641 | 0.3649 | 0.8226 | 0.0868 | 13991.4838 | 0.0396 | 1.3631* |
| 68 | -0.1553 | -0.0316 | 0.001 | 1.0701 | 0.0992 | 19681.7593 | 0.0396 | 1.3608 |
| 69 | 0.6867 | 0.1526 | 0.0234 | 1.065 | 0.0985 | 19410.5885 | 0.0471 | 1.3601 |
| 70 | 0.5235 | 0.1059 | 0.0113 | 1.0622 | 0.0988 | 19614.6484 | 0.0394 | 1.3531 |
| 71 | 0.7655 | 0.1538 | 0.0238 | 1.0523 | 0.0984 | 19638.8682 | 0.0388 | 1.3349 |
| 72 | -0.0948 | -0.0211 | 0.0005 | 1.0789 | 0.0992 | 19676.2493 | 0.047 | 1.3593 |
| 73 | -0.0644 | -0.0131 | 0.0002 | 1.0702 | 0.0992 | 19682.3367 | 0.0393 | 1.3506 |
| 74 | 0.0383 | 0.0084 | 0.0001 | 1.0792 | 0.0992 | 19656.8796 | 0.0471 | 1.3596 |

**Appendix Four: Outlier Diagnostics for Concern over mistakes**

| **Obs** | **rstudent** | **dffits** | **cook.d** | **cov.r** | **tau2.del** | **QE.del** | **hat** | **weight** |
| --- | --- | --- | --- | --- | --- | --- | --- | --- |
| 1 | 1.8054 | 0.5454 | 0.2851 | 1.0028 | 0.0964 | 3799.7427 | 0.0838 | 1.8659 |
| 2 | -0.0452 | -0.0121 | 0.0001 | 1.1179 | 0.1027 | 4029.6226 | 0.0703 | 1.8512 |
| 3 | -0.093 | -0.0229 | 0.0005 | 1.1036 | 0.1027 | 4029.4221 | 0.0586 | 1.8439 |
| 4 | -0.1262 | -0.0315 | 0.001 | 1.1056 | 0.1027 | 3988.1969 | 0.0599 | 1.885 |
| 5 | 0.6097 | 0.1533 | 0.0238 | 1.0896 | 0.102 | 4001.7979 | 0.0593 | 1.8688 |
| 6 | 1.9442 | 0.4426 | 0.1858 | 0.9473 | 0.0955 | 3459.5394 | 0.0494 | 1.8762 |
| 7 | 0.0461 | 0.0098 | 0.0001 | 1.0842 | 0.1027 | 4030.5639 | 0.0408 | 1.8807 |
| 8 | 0.1058 | 0.0219 | 0.0005 | 1.0826 | 0.1027 | 4030.3202 | 0.0402 | 1.8538 |
| 9 | -1.3976 | -0.2849 | 0.0797 | 1.0045 | 0.0989 | 3956.6241 | 0.0398 | 1.8376 |
| 10 | 1.2499 | 0.2321 | 0.0533 | 1.0125 | 0.0996 | 3834.8042 | 0.0334 | 1.8731 |
| 11 | 0.4401 | 0.0901 | 0.0082 | 1.0746 | 0.1023 | 4023.4703 | 0.04 | 1.8447 |
| 12 | 0.0741 | 0.0154 | 0.0002 | 1.0827 | 0.1027 | 4030.5184 | 0.0401 | 1.8513 |
| 13 | -1.3724 | -0.2115 | 0.044 | 0.9895 | 0.099 | 3851.685 | 0.0232 | 1.8687 |
| 14 | -1.4376 | -0.2413 | 0.0571 | 0.987 | 0.0986 | 3899.83 | 0.0274 | 1.8588 |
| 15 | 0.1737 | 0.0293 | 0.0009 | 1.0678 | 0.1027 | 4028.2097 | 0.0274 | 1.8628 |
| 16 | -0.6169 | -0.1039 | 0.0109 | 1.0535 | 0.1019 | 3959.5478 | 0.0276 | 1.8773 |
| 17 | -0.6587 | -0.1013 | 0.0104 | 1.0464 | 0.1018 | 3993.604 | 0.0231 | 1.8667 |
| 18 | 0.0165 | 0.0029 | 0 | 1.0694 | 0.1027 | 4030.5474 | 0.0276 | 1.8733 |
| 19 | -1.5759 | -0.2647 | 0.0681 | 0.9716 | 0.0978 | 3865.0262 | 0.0274 | 1.8605 |
| 20 | -0.4751 | -0.0675 | 0.0046 | 1.0507 | 0.1022 | 4025.1624 | 0.0198 | 1.8217 |
| 21 | -0.0909 | -0.0139 | 0.0002 | 1.0643 | 0.1027 | 4029.8922 | 0.0232 | 1.8743 |
| 22 | -0.6776 | -0.094 | 0.0089 | 1.0394 | 0.1017 | 4025.5124 | 0.0189 | 1.7373 |
| 23 | -0.7096 | -0.1008 | 0.0103 | 1.0396 | 0.1017 | 4017.9043 | 0.0198 | 1.8212 |
| 24 | 1.4022 | 0.2012 | 0.0397 | 0.9837 | 0.0988 | 3903.8482 | 0.0202 | 1.8574 |
| 25 | 1.2133 | 0.1677 | 0.0279 | 1.0008 | 0.0998 | 3932.858 | 0.0187 | 1.8568 |
| 26 | 1.0367 | 0.1483 | 0.022 | 1.0176 | 0.1006 | 3976.6624 | 0.0201 | 1.8475 |
| 27 | 0.1784 | 0.0256 | 0.0007 | 1.0598 | 0.1027 | 4027.1645 | 0.0202 | 1.861 |
| 28 | -0.1503 | -0.0215 | 0.0005 | 1.0601 | 0.1027 | 4029.8145 | 0.0202 | 1.8579 |
| 29 | 0.6509 | 0.0894 | 0.0081 | 1.0415 | 0.1018 | 4011.635 | 0.0185 | 1.8348 |
| 30 | -1.1288 | -0.1558 | 0.0242 | 1.0085 | 0.1002 | 3972.6106 | 0.0187 | 1.8521 |
| 31 | -1.0785 | -0.1491 | 0.0222 | 1.0128 | 0.1004 | 3969.7062 | 0.0188 | 1.8568 |
| 32 | -0.1042 | -0.0151 | 0.0002 | 1.0601 | 0.1027 | 4030.5938 | 0.0201 | 1.8416 |
| 33 | 0.5333 | 0.0734 | 0.0055 | 1.0474 | 0.1021 | 4013.4466 | 0.0187 | 1.8463 |
| 34 | 2.0791 | 0.2863 | 0.0772 | 0.9013 | 0.0946 | 3867.9386 | 0.0186 | 1.8372 |
| 35 | -1.1699 | -0.1794 | 0.032 | 1.0095 | 0.1 | 3977.7291 | 0.023 | 1.8485 |
| 36 | 0.5215 | 0.087 | 0.0077 | 1.0571 | 0.1021 | 4012.1014 | 0.0273 | 1.8436 |
| 37 | -3.7475 | -0.5753 | 0.2638 | 0.6528 | 0.08 | 2968.9184 | 0.0233 | 1.8688 |
| 38 | -0.6811 | -0.1054 | 0.0112 | 1.0456 | 0.1018 | 3988.1399 | 0.0233 | 1.873 |
| 39 | -0.843 | -0.1418 | 0.0202 | 1.04 | 0.1013 | 3998.7305 | 0.0275 | 1.8578 |
| 40 | 0.4194 | 0.0702 | 0.005 | 1.0616 | 0.1024 | 4009.7774 | 0.0275 | 1.8593 |
| 41 | 0.9062 | 0.1507 | 0.0228 | 1.0344 | 0.101 | 4000.5012 | 0.0269 | 1.8206 |
| 42 | -1.0215 | -0.1724 | 0.0297 | 1.027 | 0.1006 | 3920.3291 | 0.0277 | 1.8738 |
| 43 | 0.7185 | 0.1093 | 0.0121 | 1.042 | 0.1016 | 4010.5915 | 0.0227 | 1.8224 |
| 44 | -0.1505 | -0.0283 | 0.0008 | 1.0743 | 0.1027 | 4030.5962 | 0.0332 | 1.8535 |
| 45 | -0.3539 | -0.073 | 0.0054 | 1.0781 | 0.1025 | 4028.7745 | 0.0404 | 1.8534 |
| 46 | -0.7311 | -0.1664 | 0.028 | 1.0711 | 0.1016 | 4012.1811 | 0.0492 | 1.8579 |
| 47 | 0.0407 | 0.0098 | 0.0001 | 1.105 | 0.1027 | 4026.4194 | 0.0593 | 1.8581 |
| 48 | 0.2838 | 0.0701 | 0.005 | 1.0994 | 0.1025 | 4023.8461 | 0.0581 | 1.8204 |
| 49 | -0.2084 | -0.0528 | 0.0028 | 1.1034 | 0.1026 | 4030.5951 | 0.0593 | 1.8593 |
| 50 | 0.408 | 0.1107 | 0.0124 | 1.1082 | 0.1023 | 4021.2591 | 0.0689 | 1.8078 |
| 51 | 1.9984 | 0.5471 | 0.2835 | 0.962 | 0.0952 | 3897.487 | 0.0695 | 1.8232 |
| 52 | 0.9478 | 0.2615 | 0.0685 | 1.0798 | 0.1009 | 3948.0891 | 0.0708 | 1.8559 |
| 53 | 0.7903 | 0.2402 | 0.0581 | 1.108 | 0.1014 | 3812.757 | 0.0847 | 1.8766 |
| 54 | -0.0017 | -0.001 | 0 | 1.135 | 0.1027 | 4025.7539 | 0.084 | 1.8621 |

**Appendix Five: Outlier Diagnostics for Doubts About Actions**

| **Obs** | **rstudent** | **dffits** | **cook.d** | **cov.r** | **tau2.del** | **QE.del** | **hat** | **weight** |
| --- | --- | --- | --- | --- | --- | --- | --- | --- |
| 1 | 0.1853 | 0.0267 | 0.0007 | 1.0603 | 0.1205 | 4618.3404 | 0.0204 | 1.8762 |
| 2 | 1.0112 | 0.2554 | 0.0652 | 1.0628 | 0.1181 | 4015.2237 | 0.06 | 1.8822 |
| 3 | -0.1302 | -0.0297 | 0.0009 | 1.0934 | 0.1205 | 4601.5668 | 0.0495 | 1.873 |
| 4 | -2.3893 | -0.3697 | 0.125 | 0.8588 | 0.1081 | 3044.6107 | 0.0233 | 1.8778 |
| 5 | 0.2084 | 0.0349 | 0.0012 | 1.0672 | 0.1204 | 4615.6822 | 0.0274 | 1.8585 |
| 6 | 0.5522 | 0.0758 | 0.0058 | 1.0463 | 0.1198 | 4611.0088 | 0.0185 | 1.8319 |
| 7 | 0.1796 | 0.0301 | 0.0009 | 1.0679 | 0.1205 | 4615.353 | 0.0275 | 1.8646 |
| 8 | -2.424 | -0.4065 | 0.1511 | 0.8594 | 0.1079 | 4266.2066 | 0.0274 | 1.8535 |
| 9 | -2.1078 | -0.3244 | 0.0986 | 0.9 | 0.1107 | 4260.8207 | 0.0231 | 1.861 |
| 10 | 0.1886 | 0.026 | 0.0007 | 1.0579 | 0.1204 | 4620.1149 | 0.0187 | 1.851 |
| 11 | 0.9209 | 0.1311 | 0.0172 | 1.0262 | 0.1185 | 4598.0933 | 0.0199 | 1.8268 |
| 12 | -0.1773 | -0.0256 | 0.0007 | 1.0601 | 0.1205 | 4614.3884 | 0.0203 | 1.8676 |
| 13 | -0.1879 | -0.029 | 0.0009 | 1.0631 | 0.1205 | 4619.1251 | 0.0232 | 1.8658 |
| 14 | 0.0746 | 0.0126 | 0.0002 | 1.0694 | 0.1205 | 4620.7314 | 0.0277 | 1.8748 |
| 15 | 0.3684 | 0.076 | 0.0059 | 1.0785 | 0.1202 | 4613.6699 | 0.0408 | 1.8763 |
| 16 | 0.059 | 0.0162 | 0.0003 | 1.1193 | 0.1205 | 4612.1563 | 0.0711 | 1.8684 |
| 17 | 0.1646 | 0.0275 | 0.0008 | 1.0677 | 0.1205 | 4617.93 | 0.0274 | 1.8538 |
| 18 | -0.0127 | -0.0022 | 0 | 1.0689 | 0.1205 | 4620.0118 | 0.0274 | 1.8557 |
| 19 | -0.0858 | -0.0261 | 0.0007 | 1.1357 | 0.1205 | 4614.7623 | 0.0845 | 1.8759 |
| 20 | 0.6898 | 0.1164 | 0.0137 | 1.0497 | 0.1194 | 4555.5982 | 0.0277 | 1.8755 |
| 21 | 0.5884 | 0.0906 | 0.0083 | 1.0501 | 0.1197 | 4597.139 | 0.0232 | 1.866 |
| 22 | 0.116 | 0.0177 | 0.0003 | 1.0632 | 0.1205 | 4620.1547 | 0.0229 | 1.844 |
| 23 | -2.6505 | -0.5398 | 0.2618 | 0.8385 | 0.1058 | 4354.4852 | 0.0399 | 1.8348 |
| 24 | 0.4459 | 0.0915 | 0.0085 | 1.0751 | 0.1201 | 4616.4692 | 0.0404 | 1.8586 |
| 25 | 0.0861 | 0.0216 | 0.0005 | 1.1049 | 0.1205 | 4619.4345 | 0.0592 | 1.8594 |
| 26 | -0.5003 | -0.093 | 0.0088 | 1.0653 | 0.12 | 4563.9748 | 0.0334 | 1.8703 |
| 27 | 0.468 | 0.1418 | 0.0204 | 1.1255 | 0.12 | 4618.9164 | 0.084 | 1.8648 |
| 28 | -0.7424 | -0.1852 | 0.0346 | 1.0807 | 0.1192 | 4608.8207 | 0.0586 | 1.8391 |
| 29 | -0.6384 | -0.1754 | 0.0311 | 1.1003 | 0.1196 | 4613.3128 | 0.0702 | 1.8437 |
| 30 | 0.742 | 0.1139 | 0.0131 | 1.0415 | 0.1192 | 4582.0884 | 0.0231 | 1.8552 |
| 31 | 0.0151 | 0.0023 | 0 | 1.0647 | 0.1206 | 4620.8648 | 0.0232 | 1.8706 |
| 32 | 0.1359 | 0.0187 | 0.0004 | 1.0589 | 0.1205 | 4620.096 | 0.0188 | 1.8617 |
| 33 | 0.521 | 0.0966 | 0.0095 | 1.0641 | 0.1199 | 4588.1276 | 0.0333 | 1.8626 |
| 34 | -0.2475 | -0.0342 | 0.0012 | 1.0566 | 0.1204 | 4618.0502 | 0.0186 | 1.8446 |
| 35 | 0.15 | 0.0206 | 0.0004 | 1.0584 | 0.1205 | 4620.2578 | 0.0187 | 1.8517 |
| 36 | -0.1607 | -0.0331 | 0.0011 | 1.0826 | 0.1205 | 4621.214 | 0.0405 | 1.8618 |
| 37 | 0.694 | 0.0997 | 0.01 | 1.0414 | 0.1194 | 4589.8341 | 0.0202 | 1.8615 |
| 38 | 0.0048 | 0.001 | 0 | 1.0935 | 0.1205 | 4619.1677 | 0.0492 | 1.8615 |
| 39 | 0.1689 | 0.0241 | 0.0006 | 1.0592 | 0.1204 | 4620.9503 | 0.0199 | 1.8339 |
| 40 | 0.2523 | 0.036 | 0.0013 | 1.058 | 0.1204 | 4618.0973 | 0.02 | 1.8431 |
| 41 | -0.3362 | -0.0845 | 0.0073 | 1.1004 | 0.1203 | 4619.4386 | 0.0593 | 1.8618 |
| 42 | 0.019 | 0.0026 | 0 | 1.0572 | 0.1204 | 4621.275 | 0.0189 | 1.7382 |
| 43 | 1.3621 | 0.3736 | 0.1374 | 1.0411 | 0.1163 | 4576.666 | 0.07 | 1.8384 |
| 44 | 0.0926 | 0.0155 | 0.0002 | 1.0692 | 0.1205 | 4616.8392 | 0.0276 | 1.8731 |
| 45 | 0.3237 | 0.0885 | 0.008 | 1.1124 | 0.1203 | 4611.4937 | 0.0697 | 1.8306 |
| 46 | 0.0921 | 0.0132 | 0.0002 | 1.061 | 0.1205 | 4621.2638 | 0.0203 | 1.8661 |
| 47 | -0.6155 | -0.0849 | 0.0073 | 1.0437 | 0.1196 | 4601.1046 | 0.0187 | 1.8476 |
| 48 | 0.4571 | 0.0766 | 0.006 | 1.06 | 0.12 | 4615.2999 | 0.0273 | 1.8508 |
| 49 | 0.414 | 0.0845 | 0.0073 | 1.0753 | 0.1201 | 4619.2845 | 0.04 | 1.8366 |
| 50 | -3.4368 | -0.8597 | 0.612 | 0.7286 | 0.0975 | 4115.5708 | 0.059 | 1.8532* |
| 51 | 0.532 | 0.1087 | 0.012 | 1.071 | 0.1198 | 4615.8739 | 0.0401 | 1.8437 |
| 52 | 4.3629 | 1.3162 | 1.2875 | 0.6019 | 0.0873 | 3779.9283 | 0.0833 | 1.85* |
| 53 | 0.076 | 0.0124 | 0.0002 | 1.0655 | 0.1204 | 4620.963 | 0.0261 | 1.7715 |
| 54 | 0.064 | 0.0157 | 0.0003 | 1.1003 | 0.1204 | 4620.7728 | 0.0567 | 1.7791 |

**Appendix Six: Meta-Regression Results after Removing** **Outliers**

| **Variables** | **Model** | **estimate** | ***se*** | ***z*** | ***p*** | ***tau^2^*** | ***I^2^*** | ***R^2^*** | ***Q(df)*** |
| --- | --- | --- | --- | --- | --- | --- | --- | --- | --- |
| Perfectionism | 15 | 0.0239 | 0.0062 | 3.8392 | 0.0001 | 7.65% | 99.29% | 16.41% | 11329.4275 (70) |
| Doubts about actions | 16 | 0.0301 | 0.0076 | 3.9641 | 0.001 | 6.95% | 98.21% | 22.85% | 3333.8804 (50) |

To test the robustness of the three significant meta-regression results reported in Table 2, we identified potentially influential cases and examined whether the results changed after removing these cases. As shown in Appendices 3 to 5, we identified four potential outliers: two for perfectionism, two for doubts about actions, and none for concern over mistakes.

After removing the identified potential outliers, we re-conducted the meta-regression analyses. For perfectionism, the moderation effect remained significant, b = 0.0239, SE = 0.0062, z = 3.8392, p = .0001, τ² = 7.65%, I² = 99.29%, R² = 16.41%. For doubts about actions, the moderation effect also remained significant, b = 0.0301, SE = 0.0076, z = 3.9641, p = .001, τ² = 6.95%, I² = 98.21%, R² = 22.85%.

Compared with the original results reported in Table 2, the regression coefficients changed only modestly and remained statistically significant after removal. For perfectionism, the coefficient increased from *b* = 0.016, *SE* = 0.007, *p* = .017 to *b* = 0.0239, *SE* = 0.0062, *p* = .0001. For doubts about actions, the coefficient changed slightly from *b* = 0.032, *SE* = 0.009, *p* = .001 to *b* = 0.0301, *SE* = 0.0076, *p* = .001. Together, these findings provide additional evidence for the robustness of the significant meta-regression findings after the removal of the identified potential outliers.

**Appendix Seven: Pairwise Correlations Among Modernization Indicators**

| **Variable** | **GDP per capita** | **Urbanization** | **Divorce rates** | **Household size** | **Overall modernization** |
| --- | --- | --- | --- | --- | --- |
| GDP per capita | 1 | 0.97 | 0.84 | -0.88 | 0.91 |
| Urbanization | 0.97 | 1 | 0.9 | -0.94 | 0.94 |
| Divorce rates | 0.84 | 0.9 | 1 | -0.79 | 0.98 |
| Household size | -0.88 | -0.94 | -0.79 | 1 | -0.81 |
| Overall modernization | 0.91 | 0.94 | 0.98 | -0.81 | 1 |

**Appendix Eight: Robustness Test Using Studies without Explicitly Reported Survey Years**

| **Variables** | **Model** | **estimate** | ***se*** | ***z*** | ***p*** | ***tau^2^*** | ***I^2^*** | ***R^2^*** | ***Q(df)*** |
| --- | --- | --- | --- | --- | --- | --- | --- | --- | --- |
| Perfectionism | 17 | 0.0295 | 0.0078 | 3.7667 | 0.0002 | 0.086 | 99.42% | 19.55% | 10439.9750 (54) |

Following conventional procedures in prior cross-temporal meta-analyses, missing survey years were estimated using publication year minus two years for journal articles and defense year minus one year for master’s theses.

To evaluate the robustness of this coding procedure, we re-ran the meta-regression after excluding studies with explicitly reported survey years and retained only studies whose survey years were coded using the imputation rules. As shown in Appendix 8, the positive temporal trend remained significant and became somewhat stronger, increasing from 0.016 (SE = 0.007, z = 2.39, p = .017; k = 71, see Model 1, Table 2) to 0.0295 (SE = 0.0078, z = 3.77, p = .0002; k = 55). These findings suggest that the observed increase in perfectionism over time was unlikely to be substantially affected by the year-imputation procedure.
